# Determining optimal mulching, planting density, and nitrogen application to increase maize grain yield and nitrogen translocation efficiency in Northwest China

**DOI:** 10.1186/s12870-020-02477-2

**Published:** 2020-06-19

**Authors:** Xiukang Wang, Ge Wang, Neil C. Turner, Yingying Xing, Meitian Li, Tao Guo

**Affiliations:** 1 College of Life Sciences, Yan’an University, Yan’an, 716000 Shaanxi China; 2 The UWA Institute of Agriculture and UWA School of Agriculture and Environment, The University of Western Australia, M082, Locked Bag 5005, Perth, WA 6001 Australia

**Keywords:** Dry matter accumulation, Nitrogen apparent recovery efficiency, Nitrogen assimilation amount, Nitrogen use efficiency, Nitrogen uptake

## Abstract

**Background:**

The combination of mulch with N fertilizer application is a common agronomic technique used in the production of rainfed maize (*Zea mays L.*) to achieve higher yields under conditions of optimum planting density and adequate N supply. However, the combined effects of mulch, planting density, and N fertilizer application rate on plant N uptake and N translocation efficiency are not known. The objective of this study was to quantify the interaction effect of mulch, planting density, and N fertilizer application rate on maize grain yield, N uptake, N translocation, and N translocation efficiency. The experiment was arranged in a randomized complete block design with three factors (2 mulch levels × 2 planting densities × 4 N fertilizer application rates) replicated four times.

**Results:**

There was a significant interaction among mulch, plant density, and N fertilizer on maize grain yield, kernel number per cob, N uptake, N translocation, and N translocation efficiency. Averaged over the 3 years of the study, total plant N uptake at silking ranged from 79 to 149 kg N ha^− 1^ with no mulch and from 76 to 178 kg N ha^− 1^ with mulch. The N uptake at silking in different plant organs ranked as leaf > grain > stem > cob. Averaged across all factors, the highest N translocation was observed in leaves, which was 59.4 and 88.7% higher than observed in stems and ears, respectively. The mean vegetative organ N translocation efficiency averaged over mulch, planting density, and N fertilizer application rate treatments decreased in the order of leaf > stem > cob.

**Conclusions:**

Mulch, planting density, and N fertilizer application rate not only have significant effects on improving maize grain yield and NUE, but also on N uptake, N translocation, and N translocation efficiency. Our results showed clearly that under high planting density, the combination of mulch and moderate N fertilizer application rate was the optimal strategy for increasing maize grain yield and N use efficiency.

## Background

Two-thirds of the food consumed by the world population is provided by maize, wheat (*Triticum aestivum L.*), and rice (*Oryza sativa L.*), and these three-plant species make up the clear majority of calories in human diets [1]. Maize is one of the most important cereal crops in Northwest China [2–4]. In the future, maize production will need to continue to grow to keep pace with population growth and future dietary changes [5, 6].

Increasing maize production is highly dependent upon chemical fertilizer inputs, especially nitrogen (N) fertilizer [7]. Consequently, farmers often applied excessive amounts of N fertilizer to ensure high grain yields. This can result in huge N losses, low nitrogen use efficiency (NUE), high production costs, and environmental pollution [8–10]. The overuse of N fertilizer continues in semi-arid regions of China and the rate of N fertilizer applied by small farmers is more than 360 kg N ha^− 1^ [11] in spite of the fact that the optimal N fertilizer application amount for maize has been shown to be only 180 kg N ha^− 1^ [12–14]. Because of the serious negative effects caused by excessive fertilization, more and more attention has been given to determining optimal fertilization amounts and application methods [15–17]. Therefore, there is an urgent need to explore optimal field management options to improve grain yield and NUE, and to inform farmers about these options.

The combination of plastic film mulch and reasonable amounts of N fertilizer application can significantly increase maize grain yield in Northwest China [13]. Plastic mulch is widely used in global agricultural production. Plastic mulch is used on 20% of the cultivated land area in China [18]. Plastic mulch has been widely used in maize production in Northwest China, and can improve the topsoil temperature and conserve soil moisture [19]. Soil nitrate-N content is a useful indicator that helps to achieve economical yield in agricultural production [20]. The application of mulch and N fertilizer has a great impact on soil nitrate-N content and thus on maize growth and yield [11, 21–23]. Compared with no mulching, film mulching significantly increased yield and NUE (unit N yield) by up to 60% [24]. Film mulching is an effective way to improve NUE and crop yield by enhancing the interactions between N fertilizer application rate, water conservation, and yield [25].

Determining reasonable and optimum planting density is an effective measure to pursue in order to achieve high yield and high NUE. The main reasons for this are that 1) an optimum population canopy structure is closely related to proper plant density [26], and 2) the full yield potential supported by existing soil fertility and precipitation is obtained with optimal planting density [27]. Planting density has proven to be a very effective agronomic strategy to improve maize grain yield [28]. The optimum planting density of commercial maize in North America is about 75,000–80,000 seeds ha^− 1^ plants under good agronomic conditions (i.e., no water or N stress), and most commercial maize breeding programs use a higher planting density (> 160,000 seeds ha^− 1^) to evaluate germplasm [29]. By contrast, the optimum maize planting density ranges from 49,850 to 65,180 seeds ha^− 1^ in China [26, 30]. Two years of field experiments showed that the highest maize grain yield was obtained when planting density was 90,000 seeds ha^− 1^ [31]. Planting density management is an important agronomic method to influence maize yields [32]. The effect of planting density on maize yield and NUE was highly correlated with plant N uptake [33]. Thus, it is necessary to optimize planting density to meet high plant N uptake.

Maize plant N uptake was mainly affected by N uptake in grain [34]. Nitrogen in maize grain has two sources: uptake from roots and transport from vegetative organs [35]. Before physiological maturity, the absorbed N cannot be transported directly to the grain, but instead is stored in vegetative organs and then transported to the grain [36, 37]. As maize approaches physiological maturity, N uptake occurs simultaneously through direct transport from the soil to the grain through root uptake and translocation of stored N from vegetative organs to grain. Leaves and stems are the main sources of grain N [38]. Although a large number of agriculturalists focus on grain yield and NUE, there is a lack of information regarding how the three factors of mulch, planting density, and N fertilizer application rate change N translocation amount and N translocation efficiency from different maize vegetative organs.

The objective of this study was to determine how plastic mulch used in conjunction with high plant population density and N fertilizer application rate can increase maize grain yield, plant N uptake, N translocation, and N translocation efficiency. We hypothesized that N translocation to grain may be significantly increased by use of plastic mulch, increased planting density, and increased N fertilizer application rate. Exploring this hypothesis can provide guidance for farmers to adopt optimal management measures and also can provide a theoretical basis for improving yield and NUE.

## Results

### Aboveground dry matter accumulation, grain yield, and yield components

**Fig. 1:**
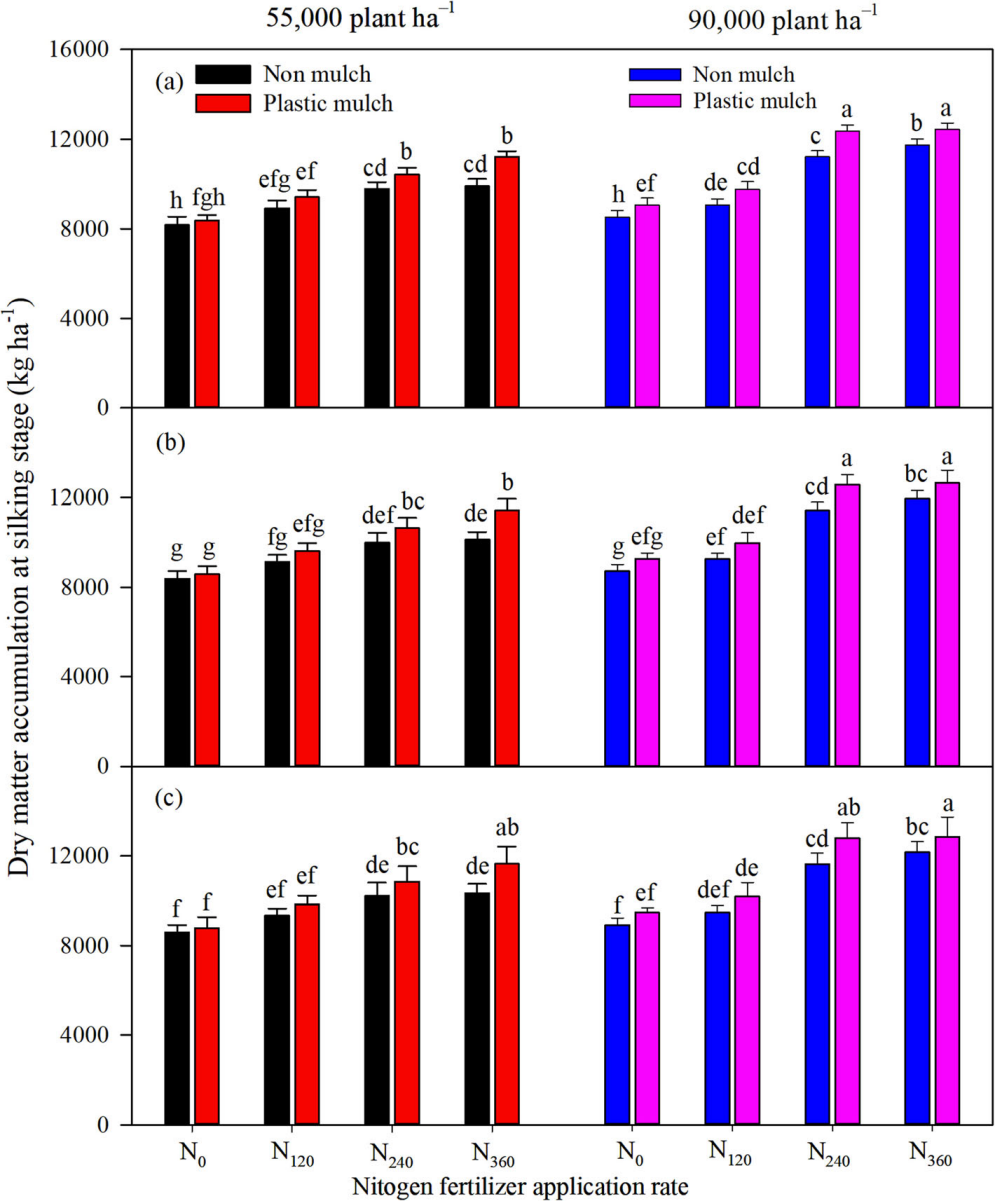
Effects of mulching (M), planting density (D), and nitrogen fertilizer application rates (N) on maize dry matter accumulation at silking in 2016 (**a**), 2017 (**b**), and 2018 (**c**). The bars represent the mean + one standard error of the mean (*n* = 4). Different letters denote significant differences between treatments (Tukey’s HSD post hoc tests, *P* < 0.05). Note: N_0_, 0 kg N ha^−1^; N_120_, 120 kg N ha^−1^; N_240_, 240 kg N ha^−1^; N_360_, 360 kg N ha^−1^

**Fig. 2:**
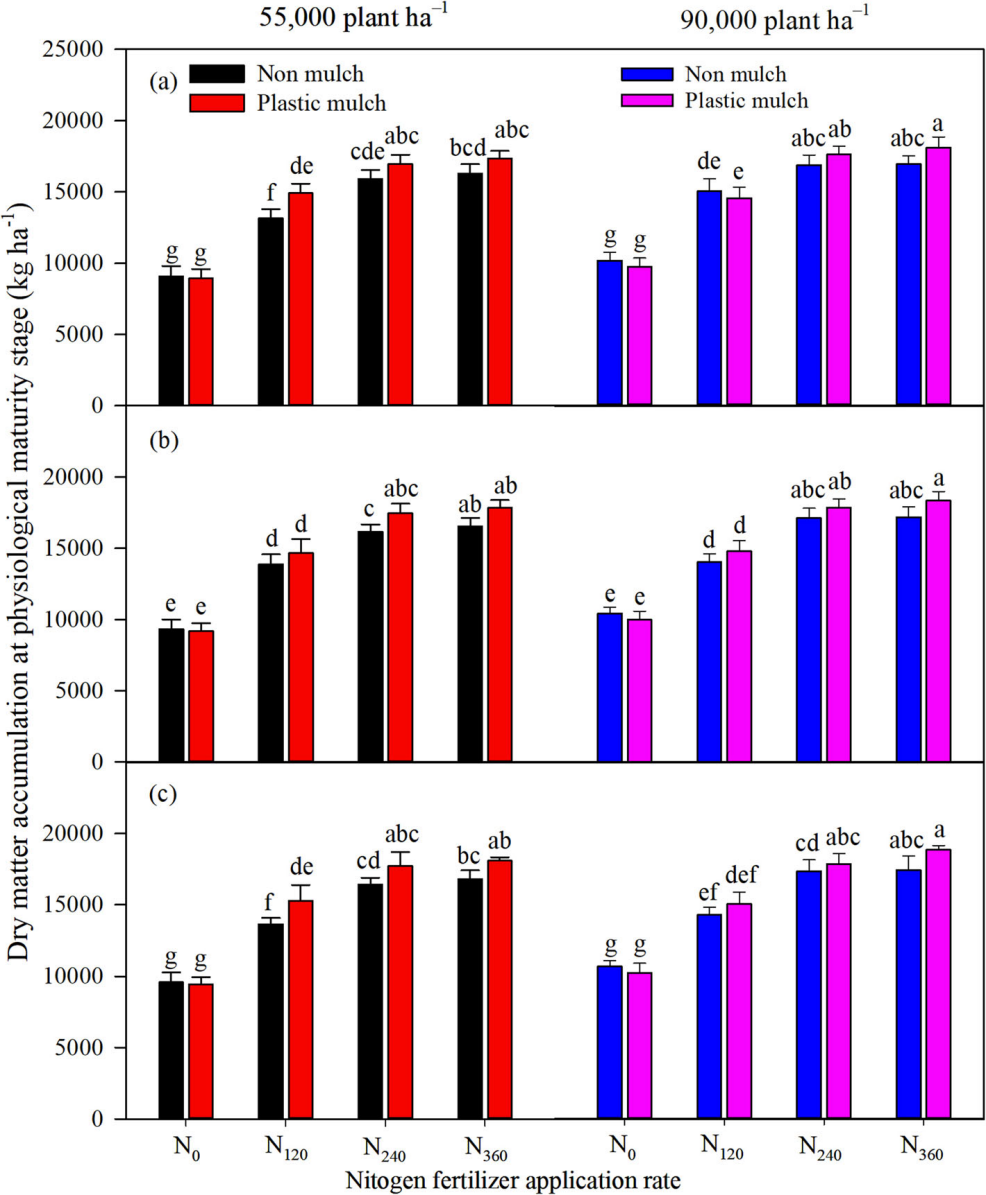
Effects of mulching (M), planting density (D), and nitrogen fertilizer application rates (N) on maize dry matter accumulation at physiological maturity in 2016 (**a**), 2017 (**b**), and 2018 (**c**). The bars represent the mean + one standard error of the mean (*n* = 4). Different letters denote significant differences between treatments (Tukey’s HSD post hoc tests, *P* < 0.05). Note: N_0_, 0 kg N ha^− 1^; N_120_,120 kg N ha^− 1^; N_240_, 240 kg N ha^− 1^; N_360_, 360 kg N ha^− 1^

The individual factors of mulch, planting density, and N fertilizer application rate significantly (*p* <  0.001) affected aboveground DMA at silking and physiological maturity, and there was a significant two-way interaction between mulch and nitrogen fertilizer application rate and a three-way interaction among the factors at silking (Table 1). The differences in DMA between different N fertilizer application rates was greater at physiological maturity than at silking stage (Figs. 1, 2). Mulching and high planting density increased maize DMA. Averaged across all N fertilizer application rates, mulch increased DMA by 6.7, 11.8, and 9.2% at physiological maturity under low planting density in 2016, 2017, and 2018, respectively, and increased DMA by 3.5, 5.3, and 4.8% under high planting density (Fig. 2). Averaged across all N fertilizer rates, DMA under high planting density was higher than DMA under low planting density with and without mulch in all 3 years (Figs. 1, 2).

**Table 1:** Treatment effects (*p* values) for dry matter accumulation (DMA), and grain yield (GY) at silking (SS) and physiological maturity (PMS), using year, mulch, nitrogen fertilizer application rates, and planting density as four fixed factors

Sources	DMA-SS	DMA-PMS	GY
Year (Y)	< 0.001	0.001	< 0.001
Mulch (M)	< 0.001	< 0.001	< 0.001
Nitrogen (N)	< 0.001	< 0.001	< 0.001
Density (D)	< 0.001	< 0.001	< 0.001
Y * M	ns	ns	ns
Y * N	ns	ns	< 0.001
Y * D	ns	ns	0.006
M * N	< 0.001	< 0.001	< 0.001
M * D	ns	0.017	< 0.001
N * D	0.001	ns	< 0.001
Y * M * N	ns	ns	ns
Y * M * D	ns	ns	ns
Y * N * D	ns	ns	ns
M * N * D	0.005	ns	< 0.001
Y * M * N * D	ns	ns	ns

Note: *ns* Not significant (*p >* 0.05)

**Fig. 3:**
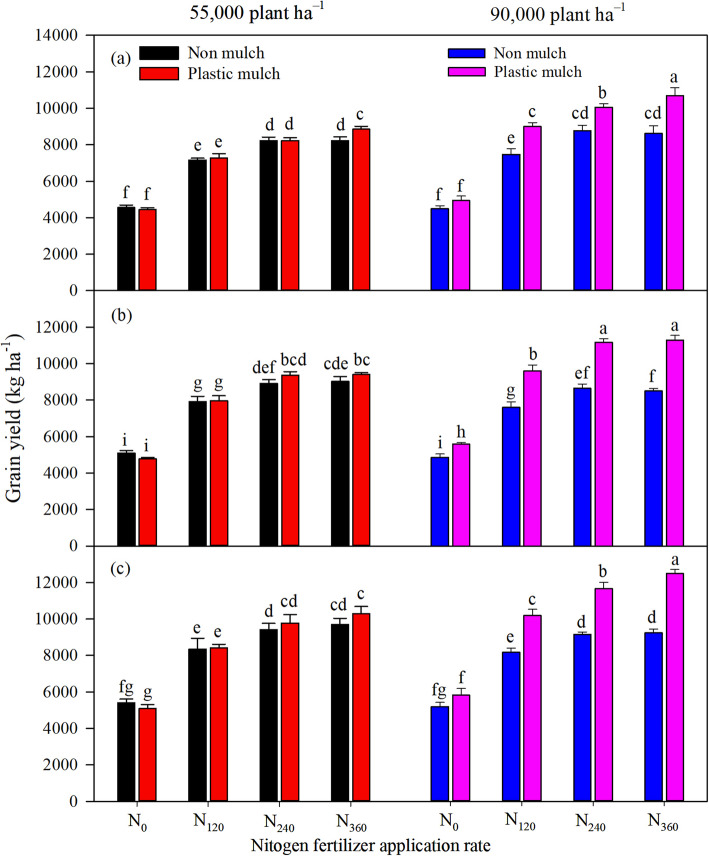
Effects of mulching, plant density, and nitrogen fertilizer application rates on maize grain yield in 2016 (**a**), 2017 (**b**), and 2018 (**c**). The bars represent the mean + one standard error of the mean (*n* = 4). Different letters denote significant differences between treatments (Tukey’s HSD post hoc tests, *P* < 0.05). Note: N_0_, 0 kg N ha^− 1^; N_120_, 120 kg N ha^− 1^; N_240_, 240 kg N ha^− 1^; N_360_, 360 kg N ha^− 1^

The individual factors of mulch, planting density, and N fertilizer application rate each had a significant influence on grain yield, and there was a significant interaction between each pair of factors. There was also a significant three-way interaction effect among these factors (Table 1). Increasing the N fertilizer application rate significantly increased grain yield (Fig. 3) under all mulch and planting density treatments, similar to what was observed for dry matter accumulation. Averaged across all N fertilizer application rates, plastic mulch increased grain yield by 4.2, 6.3, and 5.9% under the low planting density in 2016, 2017, and 2018, respectively, and increased grain yield by 20.2, 21.4, and 20.9% under high planting density (Fig. 3). Compared with low planting density, high planting density increased grain yield by 3.5, 4.4, and 5.7% under the no mulch treatment and increased grain yield by 18.1, 27.2, and 26.4% under the mulch treatment in 2016, 2017, and 2018, respectively (Fig. 3). Averaged over all 3 years, the grain yield for N_360_ (11,490 kg ha^− 1^) was the highest, which was 4.8, 19.7, and 111% higher than grain yield for N_240_, N_120_, and N_0_, respectively, with the mulch and high planting density treatments (Fig. 3).

The individual factors of mulch, planting density, and N fertilizer application rate significantly affected all yield components (Table 2) except that mulch had no significant effect on cob number. The interaction among mulch, planting density, and N fertilizer application rate had a significant effect only on kernel number per cob. Averaged across N fertilizer application rate and mulch, cob length under low planting density was 3.1, 3.6, and 4.2% higher than cob length under high planting density in 2016, 2017, and 2018, respectively (Table 2). Cob number was higher under high planting density than under low planting density under the same mulch and N fertilizer application rate treatments. Averaged across N fertilizer application rates, high planting density increased cob number by 66.9, 65.7, and 65.2% under the no mulch treatment in 2016, 2017, and 2018, respectively, and increased cob number by 65.6, 66.2, and 64.4% under mulch (Table 2). Compared with high planting density, low planting density increased kernel number per cob by 24.0, 23.2, and 21.3% under the no mulch treatment in 2016, 2017, and 2018, respectively, and by 22.8, 18.8, and 18.6% under mulch (Table 2). Because cob number was higher under high planting density, thousand kernel weight was considerably lower under high planting density than under low planting density. Compared with low planting density, high planting density decreased thousand kernel weight by 35.5, 34.0, and 36.1% under the no mulch treatment in 2016, 2017, and 2018, respectively, and by 8.8, 7.7, and 9.6% under mulch (Table 2). Averaged over planting density and N fertilizer application rate, cob length, kernel number per cob, and thousand kernel weight were higher under mulch than no mulch (Table 2).

**Table 2:** Maize cob length, cob number, kernel number per cob, and thousand kernel weight as affected by mulch, nitrogen fertilizer application rate, and planting density (D_L_: 55,000 seeds ha^− 1^; D_H_: 90,000 seeds ha^− 1^) in 2015, 2016, and 2017

Year	Mulch	Nitrogen	Cob length (cm)		Cob number (ha^− 1^)		Kernel number per cob		Thousand kernel weight (g)	
			D_L_	D_H_	D_L_	D_H_	D_L_	D_H_	D_L_	D_H_
2015	No	N_0_	14.6	15.3	42,997	77,757	470	385	223	149
		N_120_	16.6	16.3	48,703	79,281	555	437	276	211
		N_240_	17.3	16.5	48,894	79,500	571	469	315	222
		N_360_	17.2	16.5	49,023	79,953	581	465	299	240
	Yes	N_0_	15.3	14.3	45,143	76,345	473	418	214	155
		N_120_	17.2	16.8	48,642	79,087	583	461	253	248
		N_240_	17.6	16.9	49,284	81,562	617	485	271	255
		N_360_	17.5	16.8	49,112	81,349	613	497	276	265
2016	No	N_0_	15.4	15.6	45,114	77,496	484	395	234	157
		N_120_	17.0	16.5	48,892	80,244	563	447	290	221
		N_240_	17.6	16.8	49,151	79,772	579	482	330	233
		N_360_	17.4	16.6	48,679	80,270	583	469	301	252
	Yes	N_0_	15.9	15.4	45,304	77,291	478	444	225	163
		N_120_	18.0	17.1	49,151	80,691	582	459	266	261
		N_240_	18.1	17.2	49,705	82,303	613	509	285	268
		N_360_	17.8	17.2	50,029	82,422	594	495	289	278
2017	No	N_0_	16.0	15.7	44,857	78,187	492	398	246	165
		N_120_	16.9	16.7	49,651	81,211	553	448	305	232
		N_240_	17.6	16.6	49,105	79,865	576	484	347	245
		N_360_	17.4	16.5	49,166	79,301	591	494	335	264
	Yes	N_0_	15.4	15.2	45,633	76,469	484	447	236	171
		N_120_	18.0	16.9	49,764	80,041	590	468	279	274
		N_240_	17.7	17.0	49,960	81,762	615	501	299	281
		N_360_	18.1	16.9	49,391	81,838	622	525	302	292
Treatment effect (*P* values) ^a^										
Year (Y)			< 0.001		ns		0.008		< 0.001	
Mulch (M)			< 0.001		ns		< 0.001		< 0.001	
Nitrogen (N)			< 0.001		< 0.001		< 0.001		< 0.001	
Density (D)			< 0.001		< 0.001		< 0.001		< 0.001	
Y * M			ns		ns		ns		ns	
Y * N			ns		ns		ns		ns	
Y * D			ns		ns		ns		ns	
M * N			0.01		ns		ns		< 0.001	
M * D			ns		ns		ns		ns	
N * D			ns		ns		< 0.001		< 0.001	
Y * M * N			ns		ns		ns		ns	
Y * M * D			ns		ns		ns		ns	
Y * N * D			ns		ns		ns		ns	
M * N * D			ns		ns		0.006		ns	
Y * M * N * D			ns		ns		ns		ns	

^a^*ns* Not significant (*P* > 0.05)

### Nitrogen uptake at silking and at physiological maturity

**Fig. 4:**
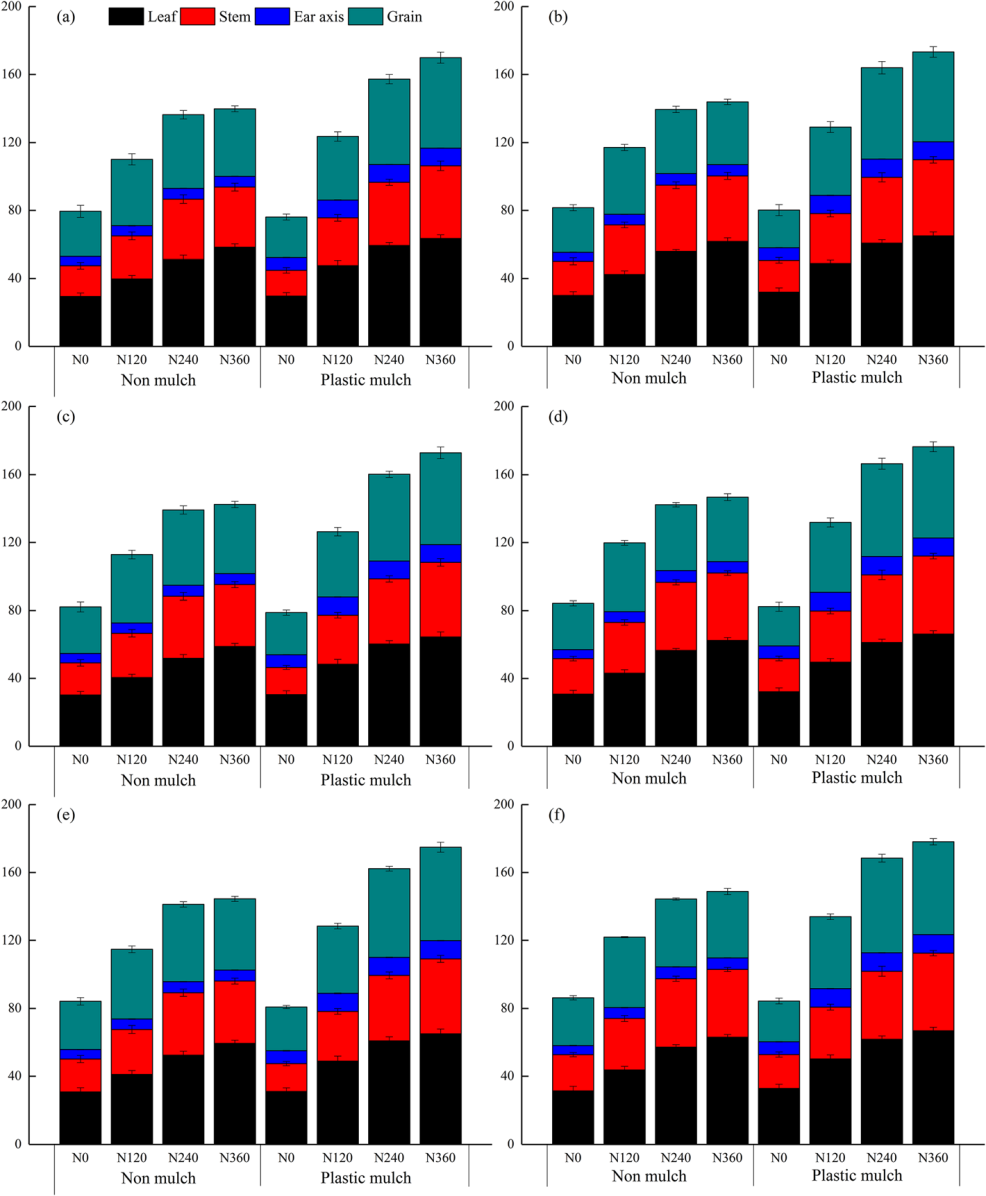
Plant nitrogen uptake at silking by different maize organs as affected by mulching (no mulch, plastic mulch), planting density (55,000 seeds ha^− 1^, 90,000 seeds ha^− 1^), and N fertilizer application rate (N_0_, 0 kg N ha^− 1^; N_120_,120 kg N ha^− 1^; N_240_, 240 kg N ha^− 1^; N_360_, 360 kg N ha^− 1^) in 2016 (**a**, **b**), 2017 (**c**, **d**), and 2018 (**e**, **f**). Bars represent the mean + one standard error of the mean (*n* = 4)

**Fig. 5:**
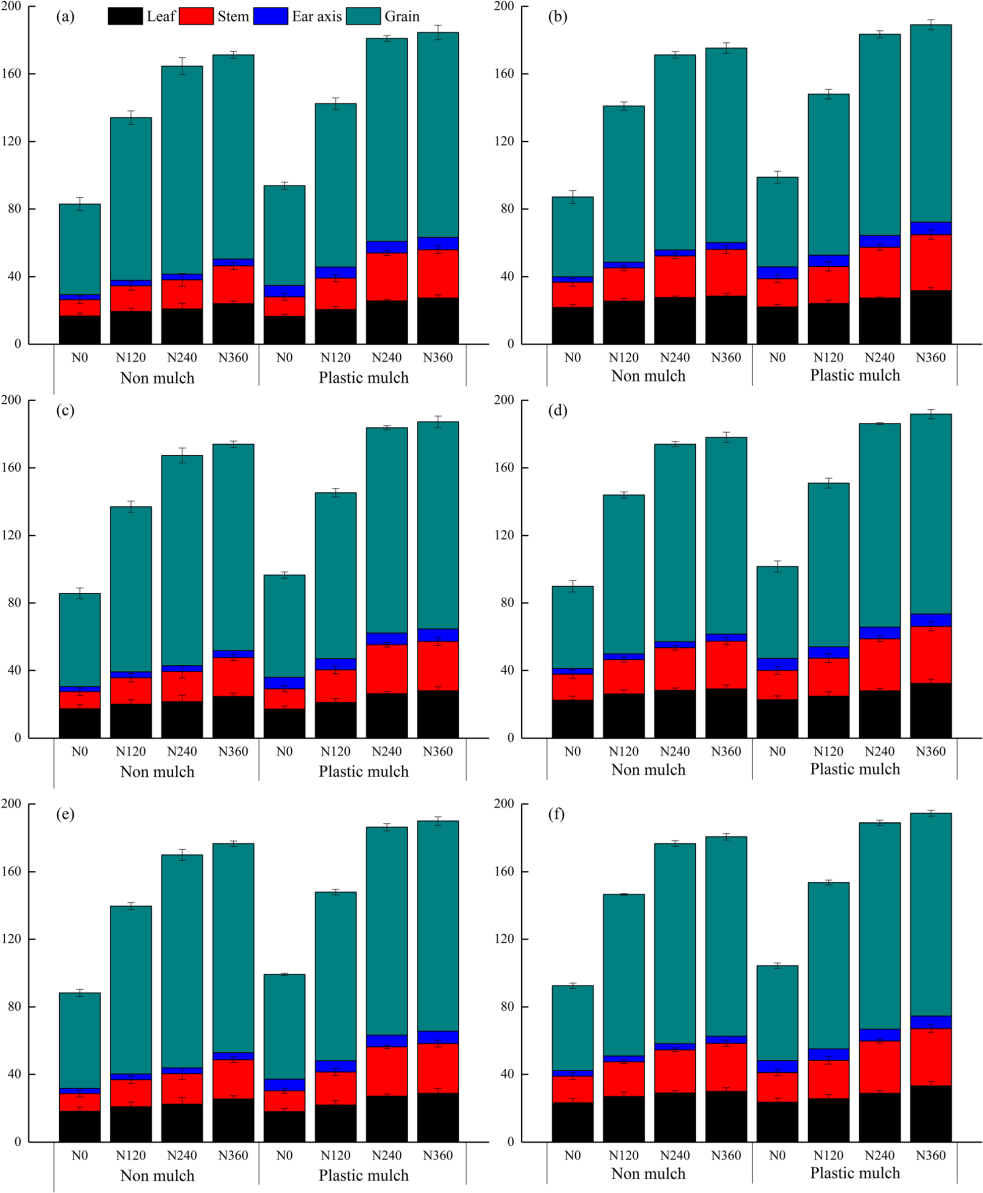
Nitrogen uptake at physiological maturity in different maize organs as affected by mulching (no mulch, plastic mulch), planting density (55,000 seeds ha^− 1^, 90,000 seeds ha^− 1^), and N fertilizer application rate (N_0_, 0 kg N ha^− 1^; N_120_, 120 kg N ha^− 1^; N_240_, 240 kg N ha^− 1^; N_360_, 360 kg N ha^− 1^) in 2015 (**a**, **b**), 2016 (**c**, **d**), and 2017 (**e**, **f**). Bars represent the mean + one standard error of the mean (*n* = 4)

Plant N uptake at silking and physiological maturity in different organs was affected by mulch, planting density, and N fertilizer application rate are shown in Figs. 4 and 5. The individual factors of mulch, planting density, and N fertilizer application rate significantly affected N uptake by different organs at silking and physiological maturity (Table 3). The interaction between mulch and N fertilizer rate had a significant effect on N uptake by different organs at silking and physiological maturity (Table 3). In addition, there was a significant three-way interaction effect among mulch, planting density, and N fertilizer rate on N uptake by different organs at silking and physiological maturity, except leaf N uptake at silking (Table 3).

**Table 3:** Treatment effects (*p* values) for the nitrogen uptake by different maize organs at silking and physiological maturity for four fixed factors (year, mulch, nitrogen fertilizer application rate, and planting density)

Sources	Nitrogen uptake at the silking				Nitrogen uptake at physiological maturity			
	Leaf	Stem	Cob	Grain	Leaf	Stem	Cob	Grain
Year (Y)	0.003	0.001	< 0.001	< 0.001	0.002	ns	< 0.001	< 0.001
Mulch (M)	< 0.001	< 0.001	< 0.001	< 0.001	< 0.001	< 0.001	< 0.001	< 0.001
Nitrogen (N)	< 0.001	< 0.001	< 0.001	< 0.001	< 0.001	< 0.001	< 0.001	< 0.001
Density (D)	< 0.001	< 0.001	< 0.001	< 0.001	< 0.001	< 0.001	< 0.001	< 0.001
Y * M	ns	ns	ns	ns	ns	ns	ns	ns
Y * N	ns	ns	ns	ns	ns	ns	ns	ns
Y * D	ns	ns	ns	ns	ns	ns	ns	ns
M * N	< 0.001	< 0.001	< 0.001	< 0.001	< 0.001	< 0.001	< 0.001	< 0.001
M * D	0.034	ns	ns	< 0.001	0.006	0.002	ns	0.001
N * D	ns	ns	< 0.001	0.007	ns	ns	< 0.001	0.012
Y * M * N	ns	ns	ns	ns	ns	ns	ns	ns
Y * M * D	ns	ns	ns	ns	ns	ns	ns	ns
Y * N * D	ns	ns	ns	ns	ns	ns	ns	ns
M * N * D	ns	0.05	0.005	< 0.001	0.01	0.008	0.007	0.028
Y * M * N * D	ns	ns	ns	ns	ns	ns	ns	ns

Note: *ns* Not significant (*p* > 0.05)

Total plant N uptake increased with increasing N fertilizer application rate (with or without mulch) at silking, and total plant N uptake was higher for mulch treatments than for no mulch treatments under the same N fertilizer application rate (Fig. 4). Averaged over the 3 years of the study, total plant N uptake at silking ranged from 79 to 149 kg N ha^− 1^ with no mulch and from 76 to 178 kg N ha^− 1^ with mulch (Fig. 4). Similarly, total plant N uptake values with mulch (ranging from 82 to 180 kg N ha^− 1^) were higher than observed with no mulch (ranging from 94 to 194 kg N ha^− 1^) at physiological maturity (Fig. 5). The N uptake by different organs at silking ranked as leaf > grain > stem > cob, but at physiological maturity the ranking changed to grain > leaf > stem > cob at physiological maturity stage (Figs. 4, 5). In general, the highest total plant N uptake was observed for the N_360_ treatment under all mulching and planting density treatments (Figs. 4, 5). Compared with the no N fertilizer (N_0_) treatment, N_120_, N_240_, and N_360_ increased total plant N uptake by 50.0, 85.8 and 95.0% at silking, respectively, and by 54.3, 90.0 and 95.6% at physiological maturity when averaged across mulch and planting density treatments and years (Figs. 4, 5). In contrast, total plant N uptake under high planting density was higher than under low planting density. Total plant N uptake was more sensitive to mulch than to planting density. Averaged across planting density, N fertilizer application rate, and years, mulch increased total plant N uptake by 13% at silking and by 8.2% at physiological maturity (Figs. 4, 5). Averaged across mulch, N fertilizer application rate, and years, high plant population density increased total plant N uptake by 3.5% at silking and by 3.4% at physiological maturity (Figs. 4, 5).

### Translocated N from vegetative organs and N translocation efficiency

**Fig. 6:**
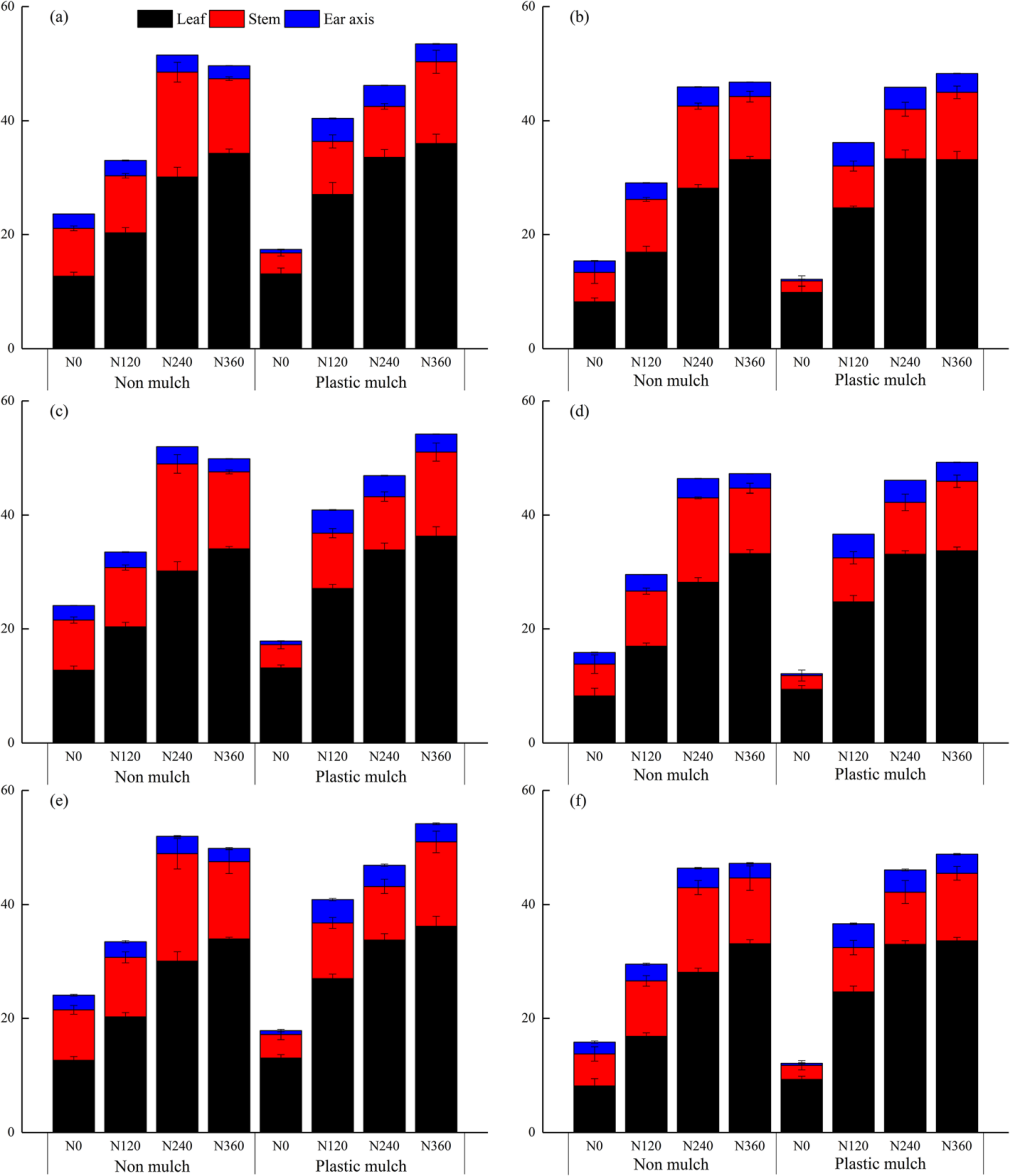
Nitrogen translocation in different maize organs (leaf, stem and cob) from silking to physiological maturity as affected by mulching (no mulch, plastic mulch), planting density (55,000 seeds ha^− 1^, 90,000 seeds ha^− 1^), and N fertilizer application rate (N_0_, 0 kg N ha^− 1^; N_120_, 120 kg N ha^− 1^; N_240_, 240 kg N ha^− 1^; N_360_, 360 kg N ha^− 1^) in 2015 (a, b), 2016 (c, d), and 2017 (e, f). Bars represent the mean + one standard error of the mean (*n* = 4)

A significant three-way interaction among mulch, planting density, and N fertilizer application rate, and significant two-way interactions between mulch and N fertilizer application rate and between planting density and N fertilizer application rate were observed for N translocation and N translocation efficiency in different maize organs. The mulch and planting density treatments had no significant interaction for N translocation and N translocation efficiency in the cob (Table 4). The individual factors of mulch, planting density, and N fertilizer application rate significantly affected N translocation and N translocation efficiency in different plant organs (Table 4). The total N translocation from vegetative organs was reduced with the increase of planting density. Total N translocation under high planting density averaged over N fertilizer application rates and years was 10.9 and 4.8% lower than observed under low planting density with and without mulch, respectively (Fig. 6). The N translocation from different organs ranked as leaf > stem > cob in all treatments (Fig. 6). Averaged across all factors, the highest N translocation was observed for leaf, which was 59.4% (14.6 kg N ha^− 1^) and 88.7% (21.9 kg N ha^− 1^) higher than that for stem and cob, respectively (Fig. 6). Leaf N translocation increased with increasing N fertilizer application rate, but N translocation in stem and cob did not follow this pattern. Averaged over mulch and planting density treatments and years, leaf N translocation for N_360_ was 8.7% (3 kg N ha^− 1^), 35.1% (12 kg N ha^− 1^), and 68.3% (23.5 kg N ha^− 1^) higher than that observed for N_240_, N_120_, and N_0_, respectively (Fig. 6).

**Table 4:** Treatment effects (*p* values) for nitrogen translocation and nitrogen translocation efficiency of different maize organs, for four fixed factors (year, mulch, nitrogen fertilizer application rate, and planting density)

Sources	Nitrogen translocation			Nitrogen translocation efficiency		
	Leaf	Stem	Cob	Leaf	Stem	Cob
Year (Y)	ns	ns	0.018	0.015	ns	ns
Mulch (M)	< 0.001	< 0.001	< 0.001	< 0.001	< 0.001	< 0.001
Nitrogen (N)	< 0.001	< 0.001	< 0.001	< 0.001	< 0.001	< 0.001
Density (D)	< 0.001	< 0.001	0.009	< 0.001	< 0.001	< 0.001
Y * M	ns	ns	ns	ns	ns	ns
Y * N	ns	ns	ns	ns	ns	ns
Y * D	ns	ns	ns	ns	ns	ns
M * N	< 0.001	< 0.001	< 0.001	< 0.001	< 0.001	< 0.001
M * D	ns	0.018	ns	< 0.001	< 0.001	ns
N * D	< 0.001	0.012	< 0.001	< 0.001	< 0.001	< 0.001
Y * M * N	ns	ns	ns	ns	ns	ns
Y * M * D	ns	ns	ns	ns	ns	ns
Y * N * D	ns	ns	ns	ns	ns	ns
M * N * D	0.001	< 0.001	0.002	0.009	0.002	< 0.001
Y * M * N * D	ns	ns	ns	ns	ns	ns

Note: *ns* Not significant (*p* > 0.05)

**Fig. 7:**
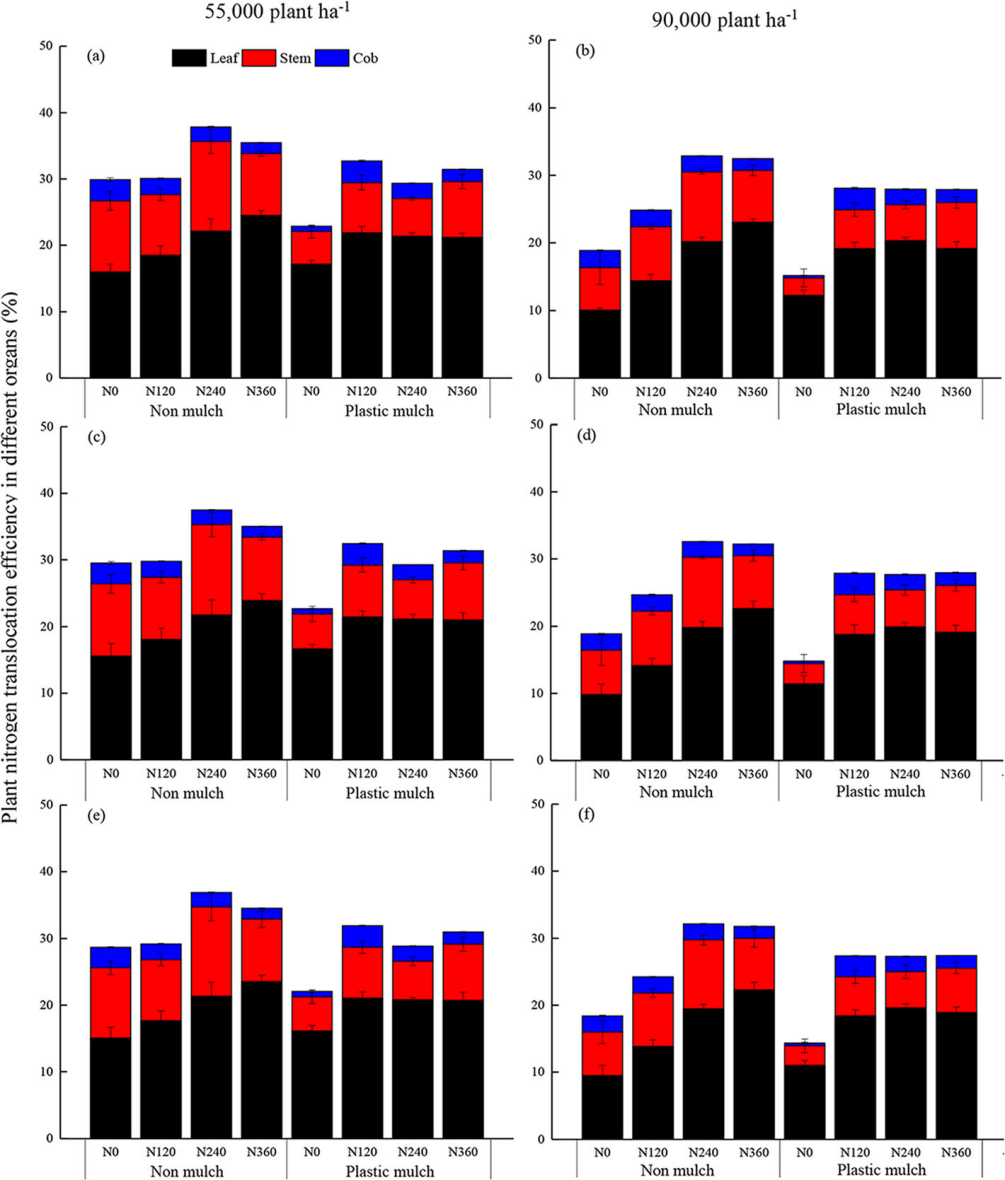
Nitrogen translocation efficiency in different maize organs (leaf, stem and cob) from silking to physiological maturity as affected by mulching (no mulch, plastic mulch), planting density (55,000 seeds ha^− 1^, 90,000 seeds ha^− 1^), and N fertilizer application rate (N_0_, 0 kg N ha^− 1^; N_120_, 120 kg N ha^− 1^; N_240_, 240 kg N ha^− 1^; N_360_, 360 kg N ha^− 1^) in 2015 (**a**, **b**), 2016 (**c**, **d**), and 2017 (**e**, **f**). Bars represent the mean + one standard error of the mean (*n* = 4)

Averaged over planting density and year, leaf N translocation efficiency increased with increasing N fertilizer application rate with the no mulch treatment, but leaf N translocation efficiency initially increased and then decreased with increasing N fertilizer application rate under the mulch treatment (Fig. 7). Considering all treatments, leaf N translocation efficiency ranged from 9.5 to 24.5%, stem N translocation efficiency ranged from 2.6 to 13.5%, and cob N translocation efficiency was ranged from 0.4 to 3.3% (Fig. 7). Averaged over N fertilizer application rate and year, plastic mulch increased leaf N translocation efficiency by 16.3% under low planting density and by 18.8% under high planting density (Fig. 7). Averaged over N fertilizer application rate and year, mulch increased leaf N translocation efficiency by 15% over the no mulch treatment, but decreased stem N translocation efficiency by 43% and decreased cob N translocation efficiency by 15.2% (Fig. 7). The mean vegetative organ N translocation efficiency averaged over mulch, planting density, and N fertilizer application rate treatments decreased in the order of leaf > stem > cob (Fig. 7).

### Nitrogen harvest index and nitrogen use efficiency

The individual factors of mulch and N fertilizer application rate significantly affected N assimilation amount (NAAS), and there was a significant interaction between mulch and N fertilizer application rate on NAAS (Table 5). The NAAS ranged from 29.9 to 74.1 kg ha^− 1^ for the low planting density and from 31.9 to 76.3 kg ha^− 1^ in the high planting density among the different treatment groups (Table 5). Averaged over mulch, planting density, and year, the highest value of NAAS was obtained with N_240_ (73.2 kg ha^− 1^), which was 49, 15.6, and 4.5% higher than NASS obtained with N_0_, N_120_, and N_360_ (Table 5).

**Table 5:** Effects of mulching, planting density (D_L_, 55,000 and D_H_, 90,000 seeds ha^− 1^), and nitrogen fertilizer application rates (N_0_, N_120_, N_240_, and N_360_; subscript indicates kg N ha^− 1^) on maize nitrogen assimilation amount after silking (NAAS), nitrogen harvest index (NHI), nitrogen use efficiency (NUE), apparent nitrogen recovery efficiency (NRE), and partial factor productivity of the fertilizer (PFP)

Year	Mulch	Nitrogen (N_kg /ha_)	NAAS (kg ha^− 1^)		NHI (%)		NUE (kg kg^− 1^)		NRE (%)		PFP (kg kg^− 1^)	
			D_L_	D_H_	D_L_	D_H_	D_L_	D_H_	D_L_	D_H_	D_L_	D_H_
2015	No	N_0_	29.9	31.9	64.7	54.2						
		N_120_	63.2	63.4	71.8	65.6	59.7	60.7	42.7	44.9	25.6	26.0
		N_240_	71.5	69.5	74.9	67.4	34.2	34.3	34	35	20.5	20.6
		N_360_	71.1	68.2	70.5	65.6	22.8	24.6	24.5	24.5	15.8	17.0
	Yes	N_0_	41.6	40.9	62.9	53.6						
		N_120_	56.3	59.2	67.9	64.4	62.2	74.9	40.5	41	26.7	32.1
		N_240_	73.9	73.1	66.3	64.9	36.5	41.9	36.3	35.2	21.9	25.1
		N_360_	67.7	68.6	65.7	61.8	24.0	29.7	25.2	25	16.6	20.5
2016	No	N_0_	31.0	42.6	64.4	54.3						
		N_120_	64.3	57.3	71.5	65.4	63.3	66.0	45.8	47.2	27.1	28.3
		N_240_	72.5	74.6	74.6	67.2	36.0	37.2	36.1	37.1	21.6	22.3
		N_360_	72.3	68.5	70.3	65.5	23.6	26.1	25.5	26.2	16.4	18.1
	Yes	N_0_	32.9	42.4	62.7	53.7						
		N_120_	64.4	60.2	67.7	64.3	66.5	80.1	43.3	44.8	28.5	34.3
		N_240_	70.5	74.4	66.1	64.7	39.0	46.6	37.1	35.2	23.4	27.9
		N_360_	69.3	69.1	65.5	61.7	25.1	31.4	26.2	25.7	17.4	21.7
2017	No	N_0_	32.6	44.3	64.2	54.4						
		N_120_	65.9	59.0	71.2	65.3	68.2	70.2	42.5	43.9	29.2	30.1
		N_240_	74.1	76.3	74.3	67.0	38.1	39.2	33.4	35.2	22.9	23.5
		N_360_	73.9	70.1	70.1	65.4	25.7	28.6	23.2	25	17.8	19.8
	Yes	N_0_	34.5	44.0	62.6	53.8						
		N_120_	66.1	61.8	67.5	64.2	69.6	84.9	41.2	41.9	29.8	36.4
		N_240_	72.1	76.0	66.0	63.4	40.8	48.6	34.2	34.7	24.5	29.1
		N_360_	70.9	71.1	63.8	61.2	27.0	34.7	24.3	24.5	18.7	24.0
Treatment effect (P values)^a^												
Year (Y)			< 0.001		ns		< 0.001		ns		< 0.001	
Mulch (M)			0.002		< 0.001		< 0.001		< 0.001		< 0.001	
Nitrogen (N)			< 0.001		< 0.001		< 0.001		< 0.001		< 0.001	
Density (D)			ns		< 0.001		< 0.001		< 0.001		< 0.001	
Y * M			ns		ns		ns		ns		ns	
Y * N			ns		ns		< 0.001		ns		0.011	
Y * D			ns		ns		0.039		ns		0.012	
M * N			< 0.001		< 0.001		< 0.001		< 0.001		< 0.001	
M * D			ns		< 0.001		< 0.001		0.01		< 0.001	
N * D			ns		< 0.001		< 0.001		0.02		0.004	
Y * M * N			ns		ns		ns		ns		ns	
Y * M * D			ns		ns		ns		ns		ns	
Y * N * D			ns		ns		ns		ns		ns	
M * N * D			ns		< 0.001		< 0.001		ns		0.004	
Y * M * N * D			ns		ns		ns		ns		ns	

^a^*ns* Not significant (*p* > 0.05)

The individual factors of mulch, planting density, and N fertilizer application rate had significant effects on N harvest index (NHI), N use efficiency (NUE), N apparent recovery efficiency (NRE), and partial factor productivity of the fertilizer (PFP) (Table 5). There was a significant three-way interaction among mulch, planting density, and N fertilizer application rate and significant two-way interactions between each pair of factors for NHI, NUE, and PFP (Table 5). The NHI for the mulch treatment was lower than observed for the no mulch treatment under the same N fertilizer application rate (Table 5). Averaged over N fertilizer application rate and year, mulch decreased NHI by 7.2% compared with the no mulch treatment for the low planting density and by 3.3% in high planting density (Table 5). However, NHI did not increase with increasing planting density. Averaged over mulch, N fertilizer application rate, and year, NHI for the high planting density was 9.3% lower than observed for the low planting density (Table 5). Averaged over N fertilizer application rate and year, mulch increased NUE by 6.5% for the low planting density and by 22% for the high planting density (Table 5). The NUE decreased with increasing N fertilizer application rate, and the N_120_ treatment had the highest NUE among all N fertilizer application rates (Table 5). Averaged over mulch treatment and year, the N_120_ treatment increased NUE by 42.3 and 62.0% over the NUE observed for the N_240_ and N_360_ treatments, respectively, at the low planting density, and by 43.4 and 60.0% at the high planting density (Table 5). Averaged over mulch, N fertilizer application rate, and year, NRE was similar for the high planting density (34.8 kg kg^− 1^) and low planting density (34.2 kg kg^− 1^), but PFP was higher for the high planting density (25.4 kg kg^− 1^) than for the low planting density (22.5 kg kg^− 1^) (Table 5). Averaged over N fertilizer application rate and planting density, mulch increased PFP by 13.9% in 2016, 14.5% in 2017, and 13.4% in 2018 compared with no mulch, (Table 5).

## Discussion

### Mulch with high planting density and increased nitrogen fertilizer application rate can increase maize grain yield and dry matter accumulation

In this study, we analyzed the influence of plastic mulch, planting density, and N fertilizer application rate and their interactions on maize aboveground dry matter accumulation and yield. This is different from previous studies that only considered the interaction of two factors on maize dry matter accumulation and yield [39–41]. In the present study, there was a significant three-way interaction among mulch, planting density, and N fertilizer and two-way interactions among each of the pairs of the three factors on maize grain yield (Table 1). The increases in maize grain yield due to mulch treatment were greater under high planting density than under low planting density. Similarly, increasing planting density can increase maize grain yield, but the effect is greater when plastic mulch is used than when no mulch is present. Maize grain yield increased with increasing N fertilizer application rate, but the rate of increase decreased with increasing N fertilizer application rate (Fig. S1). This result suggests that plastic film mulch combined with moderate N fertilizer application rate under high planting density conditions will be an optimal strategy to promote increased maize grain yield. This increase in maize yield may be related to increases in yield components (cob length, kernel number per cob, and thousand kernel weight). Although cob number increased significantly with increasing planting density, other yield components decreased with increasing planting density under the same field management [42]. This result is consistent with the previously reported finding that greater kernel number and larger kernels are important parameters for improving grain yield in areas with water and temperature limitations to growth [18].

Additionally, our findings indicated that there was a significant correlation between dry matter accumulation and maize grain yield, but the coefficient of determination was greater for the relationship between dry matter accumulation at physiological maturity and grain yield (2016, R^2^ = 0.971; 2017, R^2^ = 0.975; 2018, R^2^ = 0.964) than at silking (2016, R^2^ = 0.770; 2017, R^2^ = 0.689; 2018, R^2^ = 0.781) (Fig. S2). Therefore, the effect of dry matter accumulation after silking on maize yield is more important than dry matter accumulation before silking. This finding may be different from some previous studies that demonstrated that the period from jointing to silking is the most sensitive period for water and nutrient requirements [43, 44]. This may be due to our focus on maize grain yield (a result) while previous studies focused on maize growth (a process). Part of the reason for this phenomenon is that dry matter accumulation and N translocation play an important role between silking and physiological maturity. The source of dry matter and N translocation from organs to grain is the dry matter accumulation that occurs between jointing to silking [45–47].

While the benefits of plastic mulch have been well documented in previous studies [48–50], the present study showed that the effect of mulch on maize grain yield varied with planting density and N fertilizer application rate (Fig. 3). In three consecutive growing seasons, under low planting density, plastic mulch increased maize grain yield by 5.5% when averaged across all other factors, while under the high planting density, plastic mulch increased maize grain yield by 20.8% when averaged across all other factors. The value of mulch for increasing maize grain yield increased with increasing N fertilizer application rate under the high planting density. This result is consistent with the hypothesis that plastic mulch used in conjunction with high planting density and high N fertilizer application rate can increase maize grain yield.

In addition, the present study demonstrated that plastic mulch improved maize grain yield as planting density and N fertilizer amount increased. However, under high planting density, decreased soil evaporation is offset by increased maize transpiration [51], with the result that planting density has no significant effect on water consumption and soil water storage in arid and semi-arid regions [52, 53]. Therefore, it can be inferred that N uptake and N translocation from maize vegetative organs significantly affects maize yield under the combined action of mulch, N fertilizer application rate, and planting density.

### Maize nitrogen uptake, translocated nitrogen from vegetative organs and translocated nitrogen efficiency responses to mulch, planting density, and nitrogen fertilizer application rate

Grain N uptake at physiological maturity can be divided into two parts: 1. N assimilation from the soil, and 2. N translocated from vegetative organs [54–56]. The present study showed that mulch, planting density, and N fertilizer application rate significantly affected N uptake at silking and at physiological maturity in different plant organs, and also that there was a significant three-way interaction effect among those three factors on N uptake of different organs, except leaf N uptake at silking (Table 3). Previous studies have noted a high correlation between N uptake and crop N demand [57, 58], and a strong correlation between plant N uptake and dry matter accumulation [59]. The present study showed that there was a strong correlation between maize grain yield and N uptake (Fig. S3). This result is consistent with the results of [60] who showed that the more nitrogen absorbed and accumulated in the whole plant, the greater contribution to grain yield. The correlation coefficient between N uptake and maize grain yield varied with different vegetative organs, with higher coefficients of determination observed for leaf (Fig. S4) and stem (Fig. S5) than for cob (Fig. S6). This result suggests that maize genotype selection for increased N uptake in leaves and stems is an effective way to improve total N uptake.

Plastic mulch increases soil moisture and N uptake, thus increasing maize grain yield [61, 62]. In our study, plastic mulch increased total plant N uptake by 13% at silking and by 8.2% at physiological maturity compared with no mulch when averaged across all factors. Plastic mulch reduces evaporation losses [63], and the resultant higher soil moisture helps reduce ammonia volatilization and enhances nitrate uptake by plants [64, 65]. Moreover, converting ammonia to nitrates helps reduce reactive nitrogen emissions and maintains soil nitrogen content [7, 66]. This study demonstrated that plant N uptake under high planting density was only 3.5 and 3.4% higher than under low planting density when averaged across all factors at silking and physiological maturity, respectively. We therefore conclude that plant N uptake was more sensitive to mulch than to planting density. There may be two reasons for why high planting density increased plant N uptake. One reason may be increased utilization rate of light energy, and the other reason may be increased assimilation of soil N [67–69]. That is, the effect of promoting plant N uptake by increasing the moisture and temperature conditions of the soil with plastic mulch was greater than that of improving light utilization efficiency with increased planting density.

By comparing N uptake with N translocated, it is possible to infer that N uptake and N translocated generally balanced each other (i.e., higher N uptake corresponded to lower N translocated with no mulch and lower planting population density, Fig. 4 vs Fig. 6). With the mulch treatment, there was a clear increase in both plant N uptake and N translocated as N fertilizer application rate increased. This means that a higher proportion of plant N was retained in the vegetative organs at maturity, thereby reducing N translocation efficiency under no mulch and low planting density. Therefore, it can be concluded that the most effective way to improve NUE is to improve plant N uptake and N translocated. Previous studies have demonstrated that N translocation and accumulation are equally important to grain yield [55, 70]. In the present study, under the mulch treatment, plant N uptake increased with increasing N fertilizer application rate at silking (Fig. 4), which promoted N transfer from maize vegetative organs to kernels (Fig. 6). Together, the N uptake and N translocation increased grain N uptake at physiological maturity and improved thousand kernel weight (Table 2), ultimately resulting in high maize grain yield.

In this study, we examined the impact of mulch, planting density, and N fertilizer application rate on N translocation efficiency in maize, and found that there was a significant three-way interaction effect among the three factors on N translocation efficiency (Table 4). N translocation efficiency showed significant correlation with maize grain yield and plant N uptake (Fig. S7). However, these results did not follow the pattern of increased N translocation efficiency with increasing N fertilizer application rate (Fig. 7). Especially in the case of the no mulch treatment, the N translocation efficiency first increased and then decreased with increasing N fertilizer application rate. These results negate our hypothesis that using mulch in conjunction with high planting density can increase N translocation efficiency with increasing N fertilizer application rate. This may be due to the limitation of N uptake and storage in single maize plants. Although the N translocation efficiency did not follow the pattern of increasing with increasing N fertilizer application rate, total N uptake by maize was positively correlated with N fertilizer application rate. Adequate soil moisture and nutrients are beneficial to the growth and development of maize, which directly affect the accumulation and transport of N, and thus the total N uptake by maize [71, 72]. Previous studies have shown that increasing N fertilizer application rate can increase the N uptake before silking, and further improve N translocation from leaves and stems to grain by improving the absorption of water and nutrients by roots under better soil hydrothermal conditions (mulch vs. no mulch) [73–77]. Therefore, combining trait selection with N management research to improve N translocation efficiency is an effective way to improve NUE.

### Nitrogen harvest index and nitrogen use efficiency responses to mulch, planting density, and nitrogen fertilizer application rate

Nitrogen assimilation amount after silking (NAAS) plays an important role in increasing grain yield and reducing N residue in soil [78–81]. The present study showed that NAAS initially increased and then decreased with increasing N fertilizer application rate, and that when averaged across all factors, the highest value of NAAS was obtained with the N_240_ application rate. This result may be related to soil N availability being dependent on the physiological ability of roots to take up and assimilate N [34]. In addition, mineralized N can provide at most 50% of the N absorbed by maize during a single growing season despite extensive application of N fertilizer [82]. Planting density had no significant interaction effect on NAAS, but mulch, planting density, and N fertilizer application had a significant interaction effect on nitrogen harvest index (NHI), nitrogen use efficiency (NUE), and partial factor productivity of the fertilizer (PFP). This phenomenon may be related to the limit storing capacity of N absorbing. Vegetative organs are transformed from sink organs to source organs, and this change is accompanied by N fluxes from old tissues to young tissues and reproductive organs prior to physiological maturity [83].

During the three consecutive growing seasons of this study, the coefficient of determination for the relationship between NASS and grain yield was higher than the coefficient of determination for the relationship between NHI and grain yield (Fig. S8). It has been observed that an increase in NUE is associated with an increase in NHI, which in turn was associated with a higher N translocation efficiency in later stages of the grain filling period [84, 85]. The results of our study demonstrate that NHI and N translocation efficiency have a significant linear correlation (2016, R^2^ = 0.814, *p* <  0.01; 2017, R^2^ = 0.811, *p* <  0.01; 2018, R^2^ = 0.809, *p* <  0.01) (Fig. S9). In our experiment, the higher NHI of the N_240_ treatment was associated to higher N translocation efficiency. These results partially agree with increasing planting density, and reducing N fertilizer application rate can significantly promote N absorption, such that high total N accumulation and absorption of solar radiation can result in high grain yield and NHI [42, 86].

There was a negative correlation between the N fertilizer application rate and the NUE. Reducing N fertilizer application rate can balance N supply with crop demand for N [87]. Our study also showed that the combination of mulch, high planting population density, and high N fertilizer application rate (N_360_) resulted in the highest total N uptake, but the lowest NUE. Increasing planting density can increase NUE, no matter how much N fertilizer is applied, and NUE will increase significantly under mulch treatment [88, 89]. This study showed that although NUE at N_240_ was lower than NUE at N_120_, the interaction effect between planting density and mulch was significant, which effectively improved NUE.

In order to better understand the effects of mulch, planting density, and N fertilizer application rate on crop N dynamics, NRE was calculated in this study. It has been reported that the relative importance of NRE in determining NUE varies with N fertilizer application rate [37]. The NRE is more representative of the N uptake efficiency of crops [90]. In our experiment, NRE and NUE were the same, and the highest values of both occurred at the lowest N fertilizer application rate (N_120_). However, NRE and NUE exhibited different responses to N fertilizer application rate for different planting densities and mulch treatments [25, 91]. There was a significant linear correlation between NRE and NUE and between PFP and NUE (Fig. S10). In addition, our study indicated that mulch was an effective measure to improve NRE with N application. One reason might be related to the fact that mulching increases the amount of soil heat available for maize growth during early growth stages, which has a significant impact on crop N uptake and N accumulation [92, 93]. Another reason might be a large amount of N was accumulated in maize vegetative organs under the mulch treatment at silking, which significantly increased N translocation from vegetative organs to reproductive organs during later growth stages [94]. There is no doubt that PFP decreases with increasing fertilizer application rate, and PFP was higher in mulch than with no mulch in high and low planting densities. The NUE was higher with high planting density than with low planting density under different mulch treatments and N fertilizer application rates, but NRE under high planting density was higher than under low planting density only under the no mulch treatment. Thus, the combination of increasing planting density and reducing N fertilizer application rate with mulch can better improve resource utilization by maize.

## Conclusions

In conclusion, mulch, planting density, and N fertilizer application rate not only have significant effects on improving maize grain yield and NUE, but also on N uptake, N translocation, and N translocation efficiency. Our results showed clearly that under high planting density, the combination of mulch and moderate N fertilizer application rate was the optimal strategy for increasing maize grain yield. Increasing planting density can promote N uptake and increase cob number, which results in higher grain yield and NUE at the same N fertilizer application rate. However, N translocation and N translocation efficiency did not increase with increasing N fertilizer application rate and planting density. We infer from this that approaches to improve maize grain yield and NUE should focus on a combination of field management and genetic manipulation to improve inorganic N uptake and allocation, and N translocation and its regulation. Due to excessive input of N fertilizer, the NUE of N_360_ was low. At the same time, there was no significant difference in maize grain yield when N fertilizer application rate reached a level higher than N_240_. In order to obtain relatively high grain yield and high NUE at the same time, the commonly used N fertilizer application rate (N_360_) should be reduced. Our research may be valuable for the determining environmentally friendly methods for improving N translocation to achieve efficient use of N fertilizer.

## Methods

### Plant materials and growth conditions

Seeds of maize (cv. ‘Zhengdan 958’) were obtained from the Institute of Grain Production, Henan Academy of Agricultural Sciences. All materials were grown in the field in accordance with the local legislation. The high dominance hybrid Zhengdan 958 is planted all over China. Its parental inbreeding belonged to two different heterosis groups: Zheng 58 belonged to the PA heterosis group, a subgroup of stiff stalk, and Chang 7–2 belonged to the TSPT heterosis group, a subgroup of non-stiff stalk. The field experiment was carried out during the 2016, 2017, and 2018 maize growing seasons on farms in the Northwest Loess Plateau (36°39′ N, 109°11′ E, altitude 1109 m above sea level) in Ansai District, Shaanxi Province, China. The climate at the planting site is highland continental monsoon. The average annual temperature is 8.7 °C and the mean annual rainfall is 511 mm, with about 70% of the annual rainfall occurring in the maize growing season [95]. Total rainfall at the site was measured using a rainfall recorder (wi92859, Dongxi Instrument Technology Ltd., Beijing, China). Total annual precipitation was 382, 499, and 578 mm in 2016, 2017, and 2018, respectively, and the total rainfall during the maize growing season was 256, 460, and 455 mm. The average sand, silt, and clay contents in the 0–80 cm soil profile were 15.9, 66.7, and 17.4%, respectively. Bulk density was measured by the core method, using cores that measured 3 cm in diameter, 10 cm in length, and 70.68 cm^3^ in volume. Field capacity at 33 kPa was determined using a pressure-membrane extraction apparatus. Soil organic matter was determined using the Walkley-Black method. The available phosphorus was determined using the Olsen method. Available potassium was determined by H_2_SO_4_-HCLO_4_ digestion and the molybdenum antimony-ascorbic acid colorimetric method. The soil properties of the top 80 cm were as follows: pH (1:2.5 soil: water), 8.5; bulk density, 1.28 g cm^− 3^; organic matter, 15.2 g kg^− 1^; total N, 0.77 g kg^− 1^; available phosphorus, 33.2 mg kg^− 1^; available potassium, 138.2 mg kg^− 1^; and mineral N, 13.1 mg kg^− 1^.

### Experimental design

The experiment was arranged in a randomized complete block design with three factors (2 mulch levels × 2 planting densities × 4 N fertilizer application rates) replicated four times. The two mulch levels were plastic mulch and no-mulch. Two planting densities typical for this area were used in this study (low planting density of 55,000 seeds ha^− 1^ and high planting density of 90,000 seeds ha^− 1^). The four N fertilizer application rates were 0 (N_0_), 120 (N_120_), 240 (N_240_), and 360 (N_360_) kg N ha^− 1^ applied as urea (46.4% N). The N_360_ rate is the rate commonly applied by local farmers). The plots were selected randomly with four replicates. Individual plots were 46 m^2^ (4.6 m × 10 m) and each plot was separated by a 20-cm-wide ridge as a barrier. Before mulching, each treatment received half of the total amount of N fertilizer as a basal N fertilizer application (N_0_, 0 kg ha^− 1^; N_120_, 60 kg ha^− 1^; N_240_, 120 kg ha^− 1^; N_360_, 180 kg ha^− 1^) as well as 80 kg ha^− 1^ of phosphorus pentoxide (P_2_O_5_ 44%) and 80 kg ha^− 1^ of potassium oxide (K_2_O 60%), which were applied at the same time. The other half of the N fertilizer application was applied in early July each year as top-dressing. For top-dressed N, N fertilizer was band applied in the middle of the furrow rows at a depth of 5 cm. All plots were prepared in a ridge-furrow pattern with alternating narrow space between rows (15-cm high × 40-cm wide) and wide space between rows (10-cm high × 70-cm wide). Colorless, transparent, 0.008-mm thick polyethylene film plastic mulch was purchased from the local farmers’ market, and the entire ridge surface was covered after plot preparation. Technique of the alternately planting of wide (70-cm) and narrow (40-cm) row spacing was used with plant spacing of 35 cm (55,000 seeds ha^− 1^) and 20 cm (90,000 seeds ha^− 1^). Maize (cv. ‘Zhengdan 958′) was planted on 23 April 2016, 20 April 2017, and 25 April 2018, and harvested on 12 October 2016, 8 October 2017, and 13 October 2018. After harvest, the plastic film was gathered and recycled by the manufacturer, and the soil was then plowed to a depth of 25 cm. There was no irrigation throughout the growth period.

### Plant sampling and analysis

Four adjacent plants in a row of each plot were randomly harvested at the jointing stage (JS, 7-leaf stage), tasseling stage (TS, 14-leaf stage), silking stage (SS, silks visible outside the husks), and physiological maturity stage (PMS, approximately 50 days after filling stage). The sampled plants were cut off at ground level and separated into stems (including sheaths, stems, and bract), leaves, cobs, and grains. All the plants in two randomly-selected adjacent rows of maize (rows sampled during maize silking stage are excluded) in each plot were cut off at ground level, the number of cobs counted before all the parts were oven-dried at 75 °C to constant weight. Ten ears in each plot were randomly sampled for the determination of yield components: cob number, kernel number per cob and 1000-kernel weight [96]. Then the dried samples were weighed to determine dry matter accumulation (DMA) and ground to a fine powder for N measurement. The total N concentration in each plant organ was determined using the Kjeldahl method [56].

### Calculations

The different N uptake, N translocation, and N translocation efficiency parameters were calculated following methods described by [45, 75, 97]. The plant N uptake was calculated as:

1 $$PNU=\frac{DMA\times NC}{1000}$$

where *PNU* is the plant total nitrogen uptake (kg N ha^− 1^), *DMA* is the plant dry matter accumulation (kg ha^− 1^), and *NC* is the nitrogen concentration (mg g^− 1^).

2 $$NT={PNU}_{SS}-{PNU}_{PMS}$$

where *NT* is the nitrogen translocation (kg N ha^− 1^), *PNU*_*SS*_ is the plant total nitrogen uptake of vegetative organs at silking (kg N ha^− 1^), and *PNU*_*PMS*_ is the plant total nitrogen uptake of vegetative organs at physiological maturity (kg N ha^− 1^).

3 $$NTE=\frac{NT\kern0.1em }{PNU_{SS}}\times 100$$

where *NTE* is the nitrogen translocation efficiency (%), *NT* is the nitrogen translocation (kg N ha^− 1^), and *PNU*_*SS*_ is the plant total nitrogen uptake of vegetative organs at silking (kg N ha^− 1^).

4 $$LNT={LNU}_{SS}-{LNU}_{PMS}$$

where *LNT* is the leaf nitrogen translocation (kg N ha^− 1^), *LNU*_*SS*_ is the leaf total nitrogen uptake at silking (kg N ha^− 1^), and *LNU*_*PMS*_ is the leaf total nitrogen uptake at physiological maturity (kg N ha^− 1^).

5 $$SNT={SNU}_{SS}-{SNU}_{PMS}$$

where *SNT* is the stem nitrogen translocation (kg N ha^− 1^), *SNU*_*SS*_ is the stem total nitrogen uptake at silking (kg N ha^− 1^), and *SNU*_*PMS*_ is the stem total nitrogen uptake at physiological maturity (kg N ha^− 1^).

6 $$ENT={ENU}_{SS}-{ENU}_{PMS}$$

where *ENT* is the cob nitrogen translocation (kg N ha^− 1^), *ENU*_*SS*_ is the cob total nitrogen uptake at silking (kg N ha^− 1^), and *ENU*_*PMS*_ is the cob total nitrogen uptake at physiological maturity (kg N ha^− 1^).

7 $$LNTE=\frac{LNT\kern0.1em }{PNU_{SS}}\times 100$$

where *LNTE* is the leaf nitrogen translocation efficiency (%), *LNT* is the leaf nitrogen translocation (kg N ha^− 1^), and *PNU*_*SS*_ is the plant total nitrogen uptake at silking (kg N ha^− 1^).

8 $$SNTE=\frac{SNT\kern0.1em }{PNU_{SS}}\times 100$$

where *SNTE* is the stem nitrogen translocation efficiency (%), *SNT* is the stem nitrogen translocation (kg N ha^− 1^), and *PNU*_*SS*_ is the plant total nitrogen uptake at silking (kg N ha^− 1^).

9 $$ENTE=\frac{ENT\kern0.1em }{PNU_{SS}}\times 100$$

where *ENTE* is the cob nitrogen translocation efficiency (%), *ENT* is the cob nitrogen translocation (kg N ha^− 1^) and *PNU*_*SS*_ is the plant total nitrogen uptake at silking (kg N ha^− 1^).

10 $$NAAS={GNU}_{PMS}- TNT$$

where *NAAS* is the nitrogen assimilation amount after silking (kg N ha^− 1^), *GNU*_*PMS*_ is the grain nitrogen uptake of vegetative organs at physiological maturity (kg N ha^− 1^), and *TNT* is the sum of leaves, stems, and cob nitrogen translocation (kg N ha^− 1^).

11 $$NHI=\frac{GNU\kern0.1em }{PNU\hbox{-} A}\times 100$$

where *NHI* is the nitrogen harvest index (%), *GNU* is the grain total nitrogen uptake at physiological maturity (kg N ha^− 1^), and *PNU-A* is the total nitrogen uptake by aboveground organs at physiological maturity (kg N ha^− 1^).

12 $$NUE=\frac{GY\kern0.1em }{N_{\mathrm{amount}}}$$

where *NUE* is the nitrogen use efficiency (kg kg^− 1^), *GY* is the grain yield (kg ha^− 1^), and *N*_amount_ is the nitrogen fertilizer application amount (kg N ha^− 1^).

13 $$NRE=\frac{PNU\hbox{-} {PMS}_F- PNU\hbox{-} {PMS}_Z\kern0.1em }{N_{\mathrm{amount}}}\times 100$$

where *NRE* is the nitrogen apparent recovery efficiency (%), *PNU-PMS*_*F*_ is the plant total nitrogen uptake in the nitrogen fertilizer application plot at physiological maturity (kg N ha^− 1^), and *PNU-PMS*_*Z*_ is the plant total nitrogen uptake in the no fertilization plot at physiological maturity (kg ha^− 1^).

14 $$PFP=\frac{GY\kern0.1em }{N_{\mathrm{amount}}+{P}_{\mathrm{amount}}+{K}_{\mathrm{amount}}}$$

where *PFP* is the partial factor productivity of the fertilizer (kg ha^− 1^), *GY* is the grain yield (kg ha^− 1^), *N*_amount_ is the amount of nitrogen fertilizer application (kg N ha^− 1^), *P*_amount_ is the amount of phosphate fertilizer application (kg P_2_O_5_ ha^− 1^), and *K*_amount_ is the amount of potassium fertilizer application (kg K_2_O ha^− 1^).

### Statistical analysis

Three-way analysis of variance (ANOVA), with N application rates, plastic mulch, and planting density as the three fixed factors, was used to assess variations in each indicator. Differences between all treatments were detected using least significant difference (LSD) testing at the 0.05 significance level. We compared each indicator by use of one-way ANOVA, followed by post-hoc pairwise comparisons (Tukey’s Honestly Significant Difference [HSD] procedure), if warranted. Statistical analyses and data plotting were performed using SPSS Statistics Software 16.0 (https://www.ibm.com/products/spss-statistics) and Sigma Plot 14.0 https://systatsoftware.com/products/sigmaplot/), respectively.

## Supplementary information

**Additional file 1: Figure S1.** The relationship between nitrogen fertilizer application rate and maize grain yield in low plant population density and high plant population density in 2016, 2017, and 2018. **Figure S2.** The relationship between aboveground dry matter accumulation and maize grain yield in 2016, 2017, and 2018. **Figure S3.** The relationship between plant total nitrogen uptake and maize grain yield 2016, 2017, and 2018. **Figure S4.** The relationship between leaf nitrogen uptake and maize grain yield in 2016, 2017, and 2018. **Figure S5.** The relationship between stem nitrogen uptake and maize grain yield in 2016, 2017, and 2018. **Figure S6.** The relationship between ear axis nitrogen uptake and maize grain yield in 2016, 2017, and 2018. **Figure S7.** The relationship between translocated nitrogen efficiency and grain yield and translocated nitrogen efficiency and plant nitrogen uptake in 2016, 2017, and 2018. **Figure S8.** The relationship between nitrogen assimilation amount after silking stage and grain yield and nitrogen harvest index and grain yield in 2016, 2017, and 2018. **Figure S9.** The relationship between translocated nitrogen and nitrogen harvest index and translocated nitrogen efficiency and nitrogen harvest index in 2016, 2017, and 2018. **Figure S10.** The relationship between nitrogen use efficiency and apparent nitrogen use efficiency and nitrogen use efficiency and partial factor productivity of the fertilizer in 2016, 2017, and 2018.

## Data Availability

Datasets used in the current study are available from the corresponding author on reasonable request.

## Abbreviations

N: Nitrogen
NUE: Nitrogen use efficiency
PA: Group A germplasm derived from modern U.S. hybrids
TSPT: Tangsipingtou
JS: Jointing stage
TS: Tasseling stage
SS: Silking stage
PMS: Physiological maturity stage
DMA: Dry matter accumulation
PNU: Plant total nitrogen uptake
NC: Nitrogen concentration
NT: Nitrogen translocation
PNU_SS_: Plant total nitrogen uptake of vegetative organs at silking
PNU_PMS_: Plant total nitrogen uptake of vegetative organs at physiological maturity
NTE: Nitrogen translocation efficiency
LNT: Leaf nitrogen translocation
LNU_SS_: Leaf total nitrogen uptake at silking
LNU_PMS_: Leaf total nitrogen uptake at physiological maturity
SNT: Stem nitrogen translocation
SNU_SS_: Stem total nitrogen uptake at silking
SNU_PMS_: Stem total nitrogen uptake at physiological maturity
ENT: Cob nitrogen translocation
ENU_SS_: Cob total nitrogen uptake at silking
ENU_PMS_: Cob total nitrogen uptake at physiological maturity
LNTE: Leaf nitrogen translocation efficiency
ENTE: Cob nitrogen translocation efficiency
NAAS: Nitrogen assimilation amount after silking
GNU_PMS_: Grain nitrogen uptake of vegetative organs at physiological maturity
TNT: Sum of leaves, stems, and cob nitrogen translocation
NHI: Nitrogen harvest index
GNU: Grain total nitrogen uptake at physiological maturity
PNU-A: Total nitrogen uptake by aboveground organs at physiological maturity
GY: Grain yield
N_amount_: Nitrogen fertilizer application amount
NRE: Nitrogen apparent recovery efficiency
PNU-PMS_F_: Plant total nitrogen uptake in the nitrogen fertilizer application plot at physiological maturity
PNU-PMS_Z_: Plant total nitrogen uptake in the no fertilization plot at physiological maturity
PFP: Partial factor productivity of the fertilizer
P_amount_: Amount of phosphate fertilizer application
K_amount_: Amount of potassium fertilizer application
ANOVA: Analysis of variance
LSD: Least significant difference
HSD: Honestly significant difference
