# Supplementary material for: Determining optimal mulching, planting density, and nitrogen application to increase maize grain yield and nitrogen translocation efficiency in Northwest China
Source: BMC Plant Biol. 2020 Jun 19;20:282. doi: 10.1186/s12870-020-02477-2 (PMC7304207; doi:10.1186/s12870-020-02477-2)
Supplement: Supplementary file 1 — Additional file 1: Figure S1. The relationship between nitrogen fertilizer application rate and maize grain yield in low plant population density and high plant population density in 2016, 2017, and 2018. Figure S2. The relationship between aboveground dry matter accumulation and maize grain yield in 2016, 2017, and 2018. Figure S3. The relationship between plant total nitrogen uptake and maize grain yield 2016, 2017, and 2018. Figure S4. The relationship between leaf nitrogen uptake and maize grain yield in 2016, 2017, and 2018. Figure S5. The relationship between stem nitrogen uptake and maize grain yield in 2016, 2017, and 2018. Figure S6. The relationship between ear axis nitrogen uptake and maize grain yield in 2016, 2017, and 2018. Figure S7. The relationship between translocated nitrogen efficiency and grain yield and translocated nitrogen efficiency and plant nitrogen uptake in 2016, 2017, and 2018. Figure S8. The relationship between nitrogen assimilation amount after silking stage and grain yield and nitrogen harvest index and grain yield in 2016, 2017, and 2018. Figure S9. The relationship between translocated nitrogen and nitrogen harvest index and translocated nitrogen efficiency and nitrogen harvest index in 2016, 2017, and 2018. Figure S10. The relationship between nitrogen use efficiency and apparent nitrogen use efficiency and nitrogen use efficiency and partial factor productivity of the fertilizer in 2016, 2017, and 2018. [file 12870_2020_2477_MOESM1_ESM.doc]

**Supplementary Information (SI)**

**Manuscript title:**

Determining optimal mulching, planting density, and nitrogen application to increase maize grain yield and nitrogen translocation efficiency in northwest China

**Manuscript type:**

RESEARCH ARTICLE

**Authors:**

Xiukang Wang^1*^, Ge Wang^1^, Neil C. Turner^2^, Yingying Xing^1*^, Meitian Li^1^, Tao Guo^1^

**Author affiliations:**

^1^ College of Life Sciences, Yan'an University, Yan'an, Shaanxi 716000, China

^2^ The UWA Institute of Agriculture and UWA School of Agriculture and Environment, The University of Western Australia, M082, Locked Bag 5005, Perth, WA 6001, Australia.

**Corresponding author:**

College of Life Sciences, Yan'an University, Yan'an, Shaanxi 716000, China

wangxiukang@126.com (Xiukang Wang); xingyingying610624@163.com (Yingying Xing)


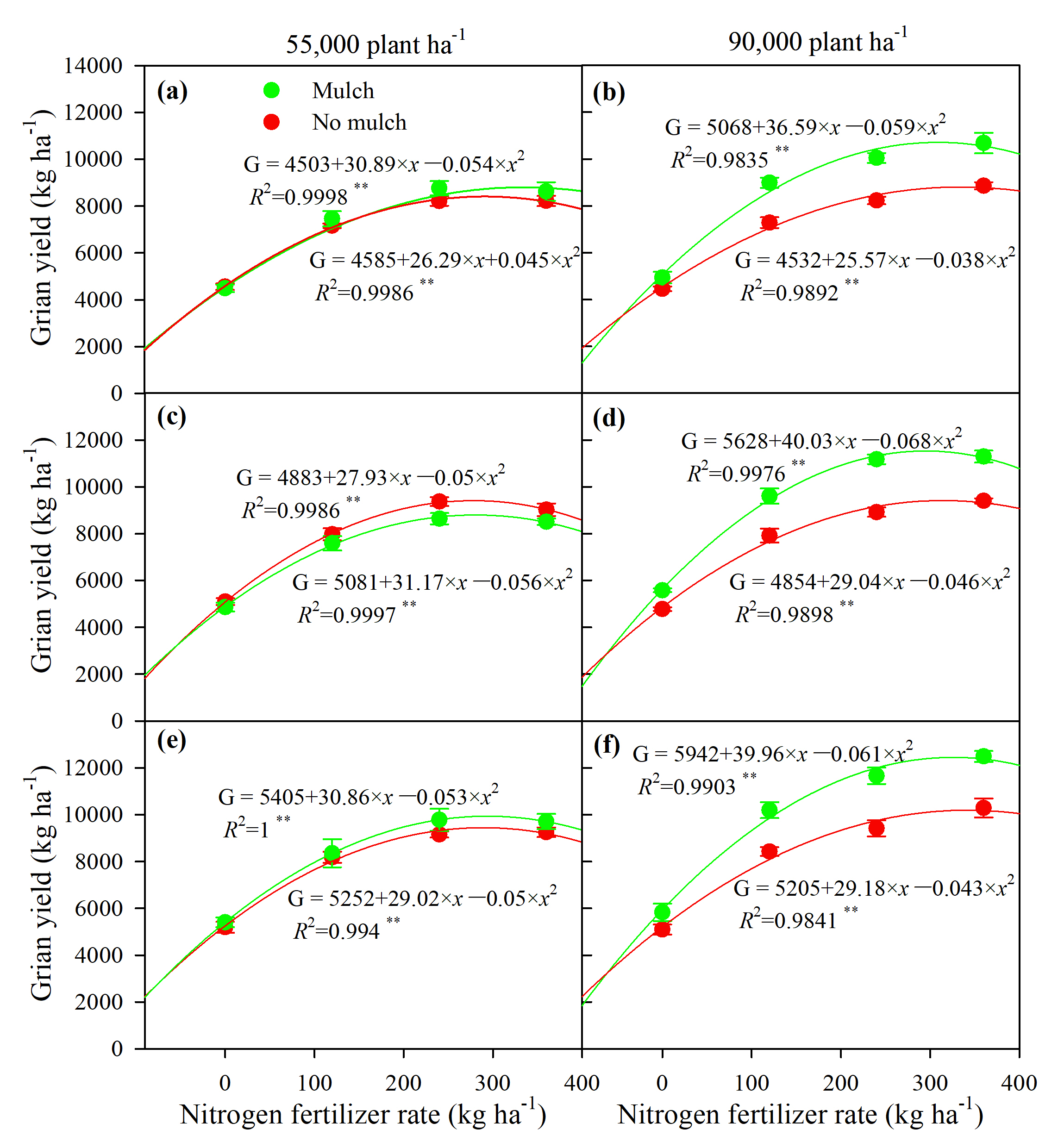


Figure S1. The relationship between nitrogen fertilizer application rate and maize grain yield in low plant population density (55,000 plant ha^-1^, a, c, e) and high plant population density (90,000 plant ha^-1^, b, d, f) in 2016 (a, b), 2017 (c, d), and 2018 (e, f). Note: N_0_, 0 kg N ha^–1^; N_120_,120 kg N ha^–1^; N_240_, 240 kg N ha^–1^; N_360_, 360 kg N ha^–1^. “**” means p < 0.01, “*” means 0.05 < p < 0.01 and “ns” means p > 0.05.


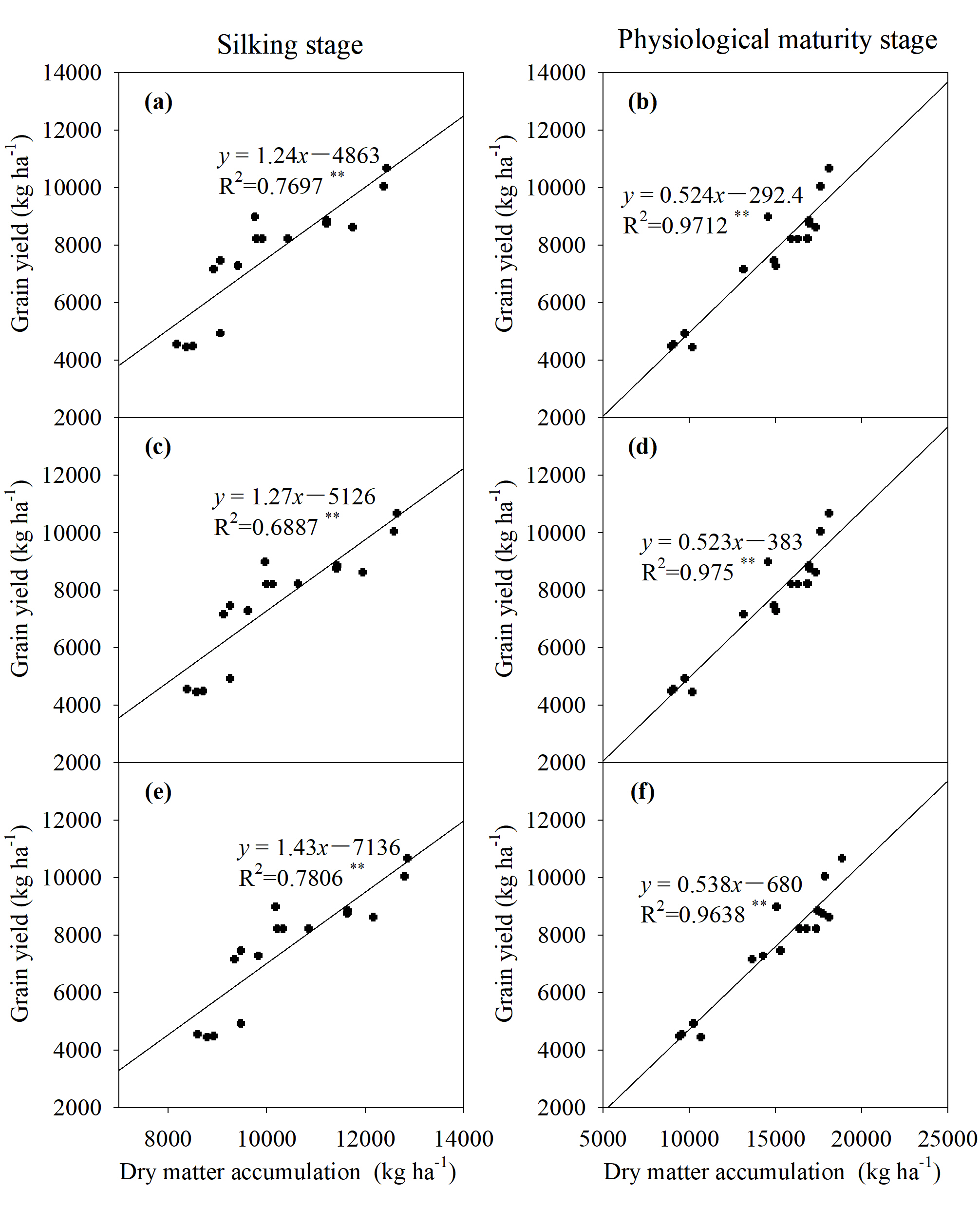


Figure S2. The relationship between aboveground dry matter accumulation and maize grain yield in 2016 (a, b), 2017 (c, d), and 2018 (e, f). “**” means p < 0.01, “*” means 0.05 < p < 0.01 and “ns” means p > 0.05.


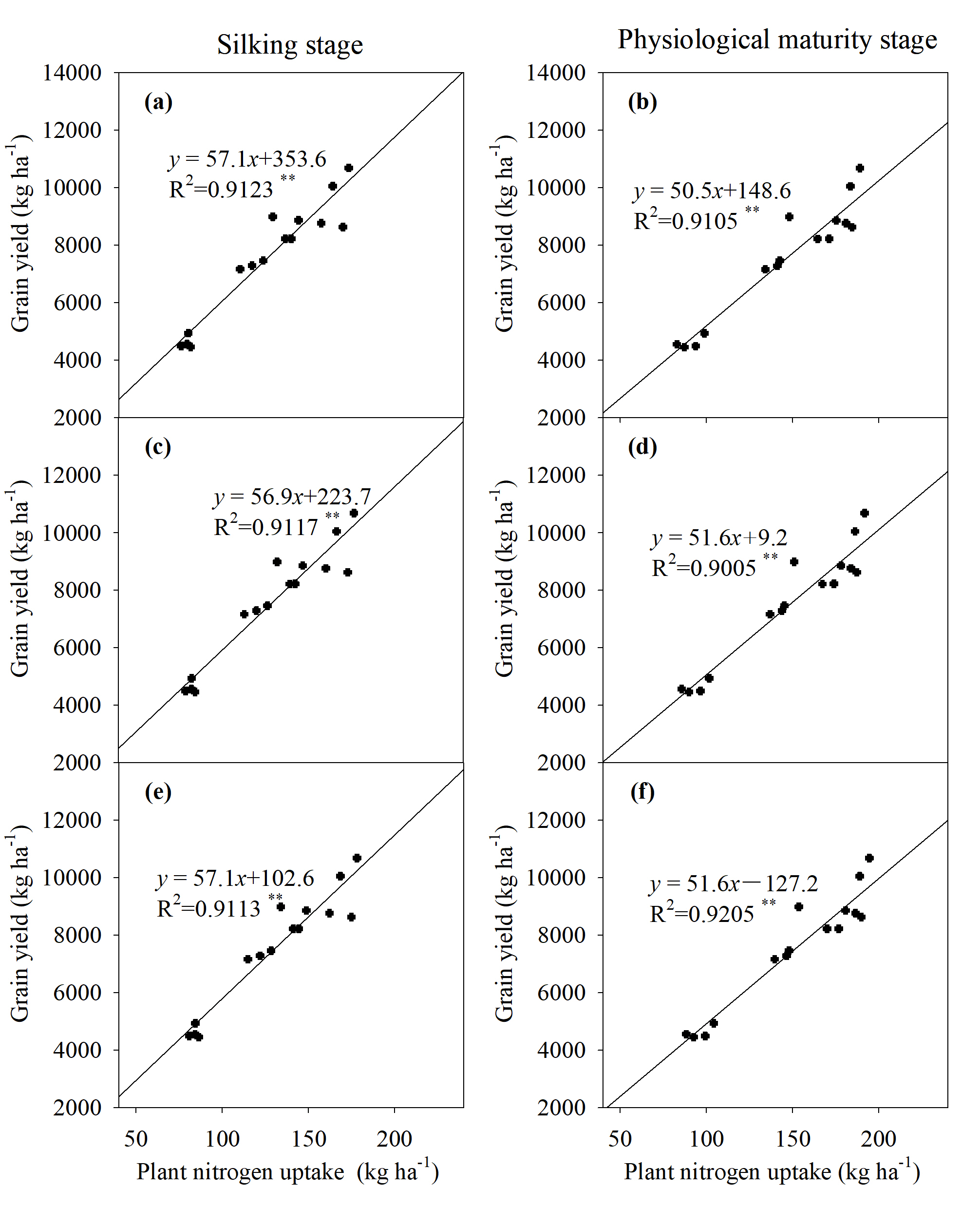


Figure S3. The relationship between plant total nitrogen uptake and maize grain yield in 2016 (a, b), 2017 (c, d), and 2018 (e, f). “**” means p < 0.01, “*” means 0.05 < p < 0.01 and “ns” means p > 0.05.


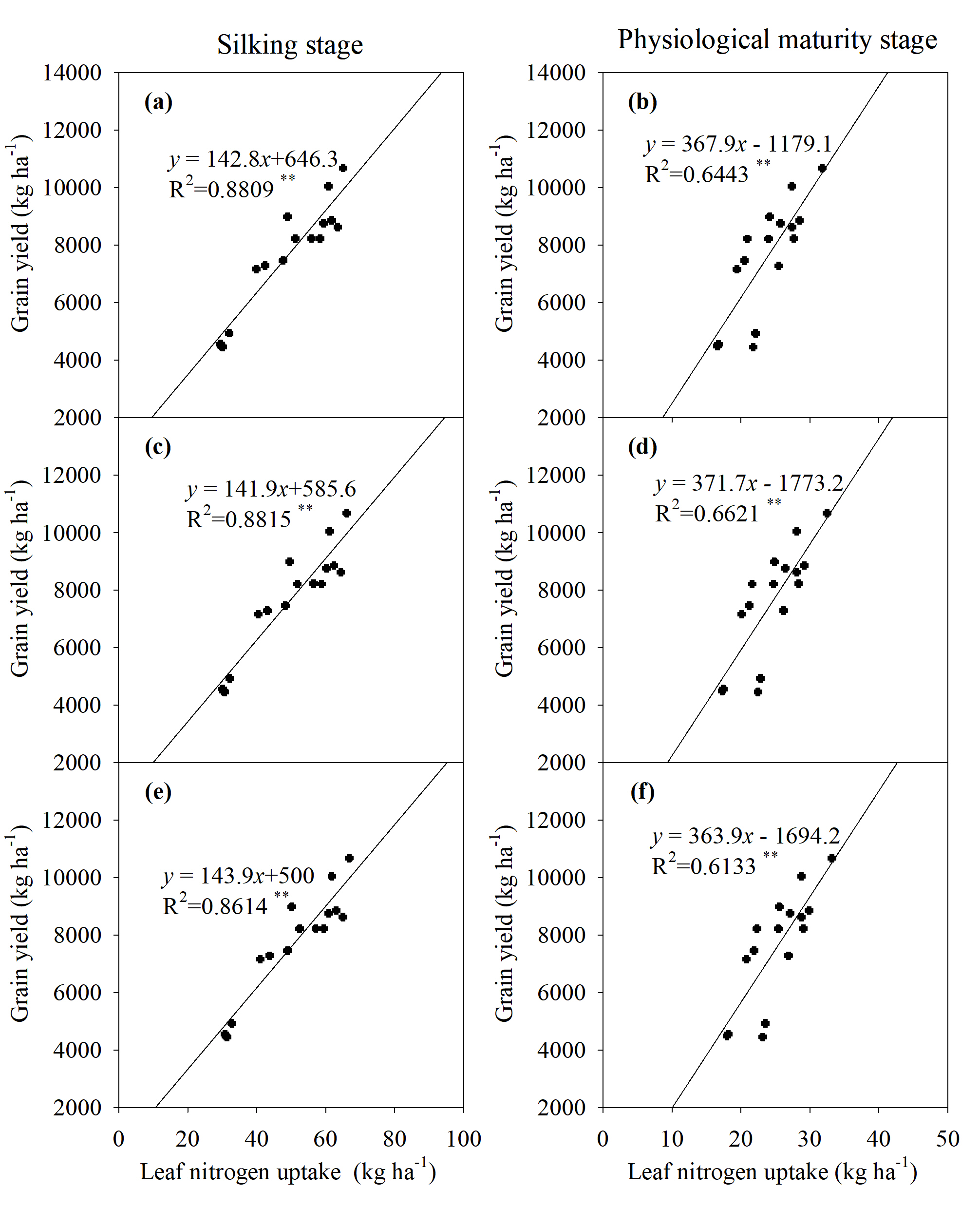


Figure S4. The relationship between leaf nitrogen uptake and maize grain yield in 2016 (a, b), 2017 (c, d), and 2018 (e, f). “**” means p < 0.01, “*” means 0.05 < p < 0.01 and “ns” means p > 0.05.


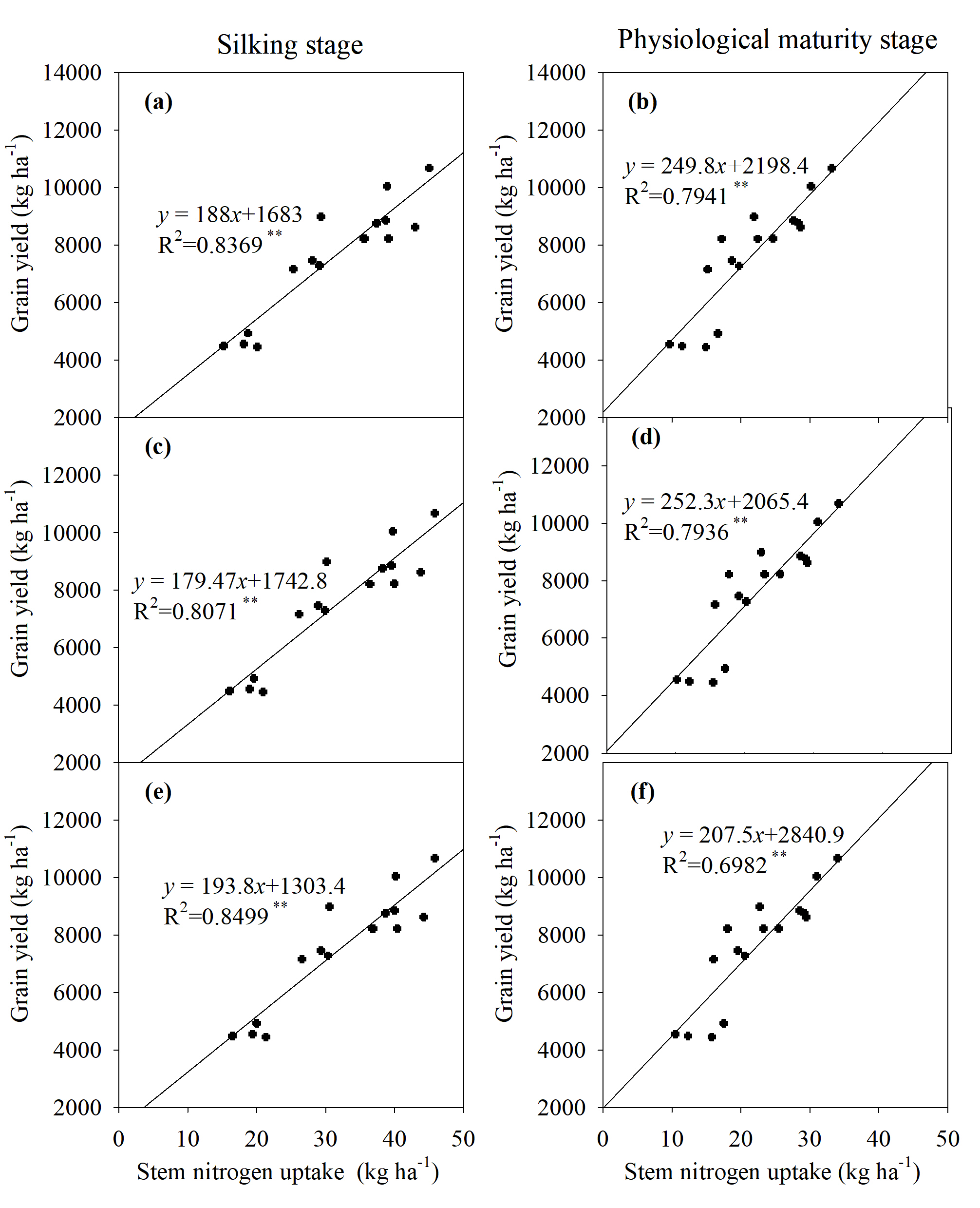


Figure S5. The relationship between stem nitrogen uptake and maize grain yield in 2016 (a, b), 2017 (c, d), and 2018 (e, f). “**” means p < 0.01, “*” means 0.05 < p < 0.01 and “ns” means p > 0.05.


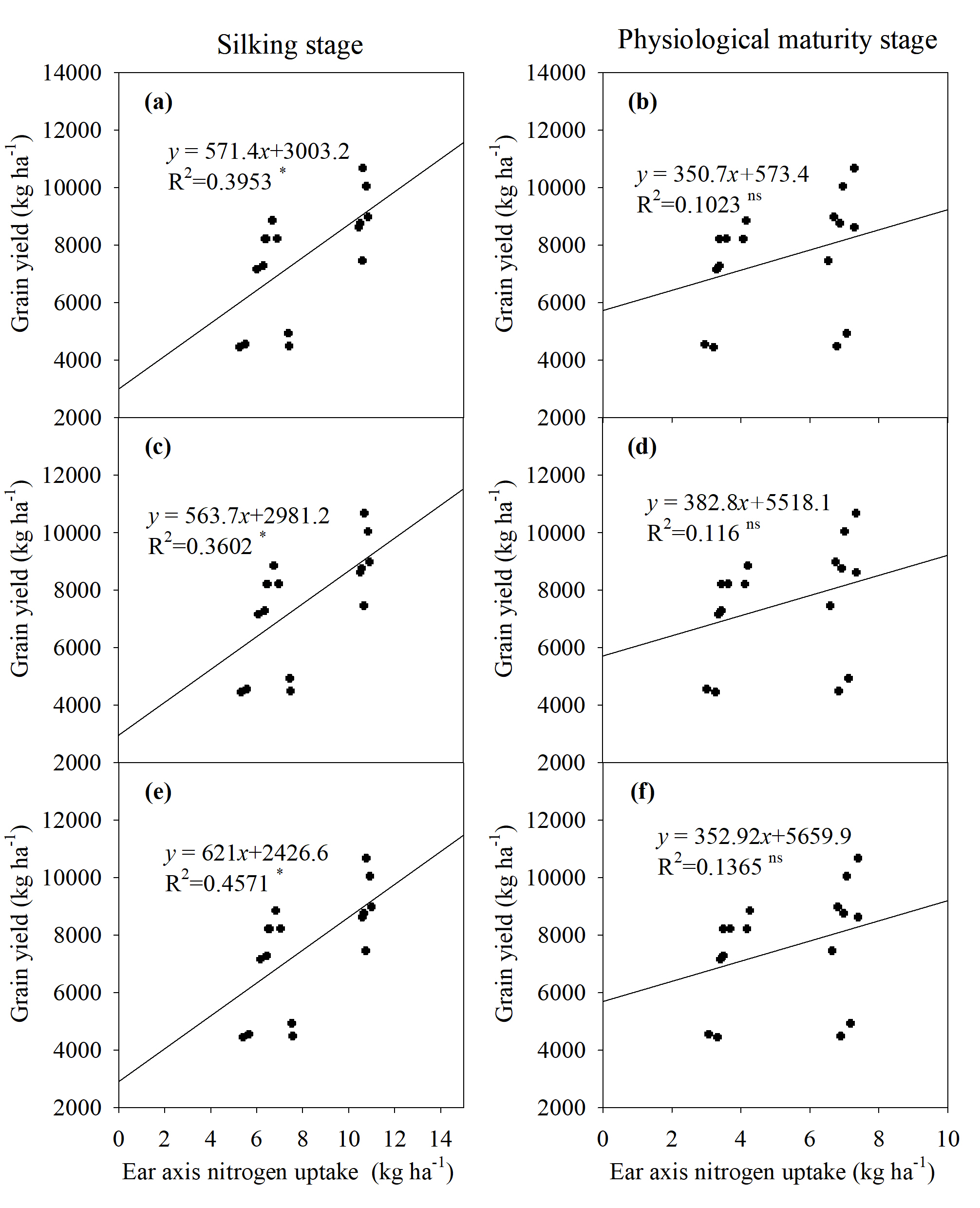


Figure S6. The relationship between ear axis nitrogen uptake and maize grain yield in 2016 (a, b), 2017 (c, d), and 2018 (e, f). “**” means p < 0.01, “*” means 0.05 < p < 0.01 and “ns” means p > 0.05.


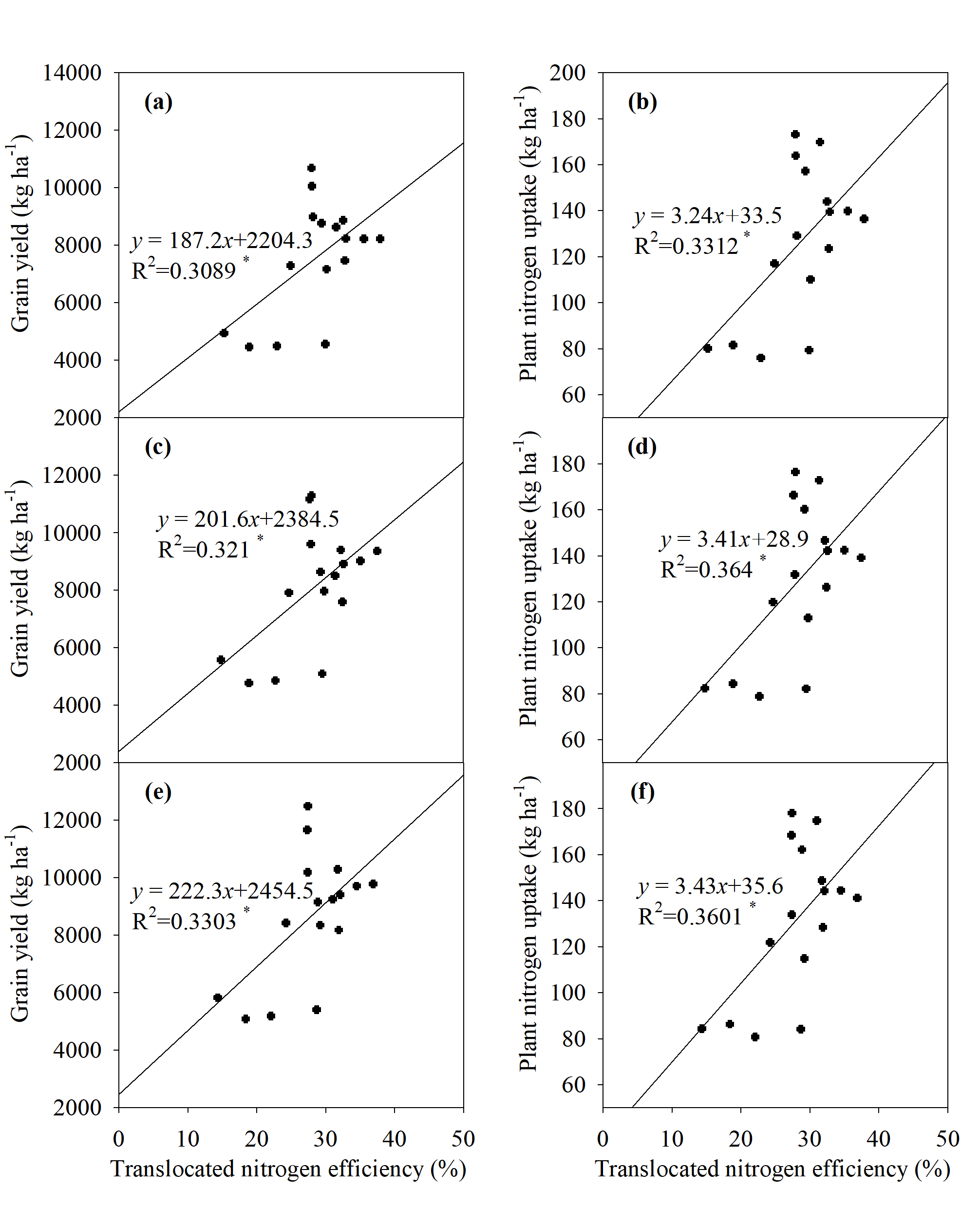


Figure S7. The relationship between translocated nitrogen efficiency and grain yield (a, c, e) and translocated nitrogen efficiency and plant nitrogen uptake (b, d, f) in 2016 (a, b), 2017 (c, d), and 2018 (e, f). “**” means p < 0.01, “*” means 0.05 < p < 0.01 and “ns” means p > 0.05.


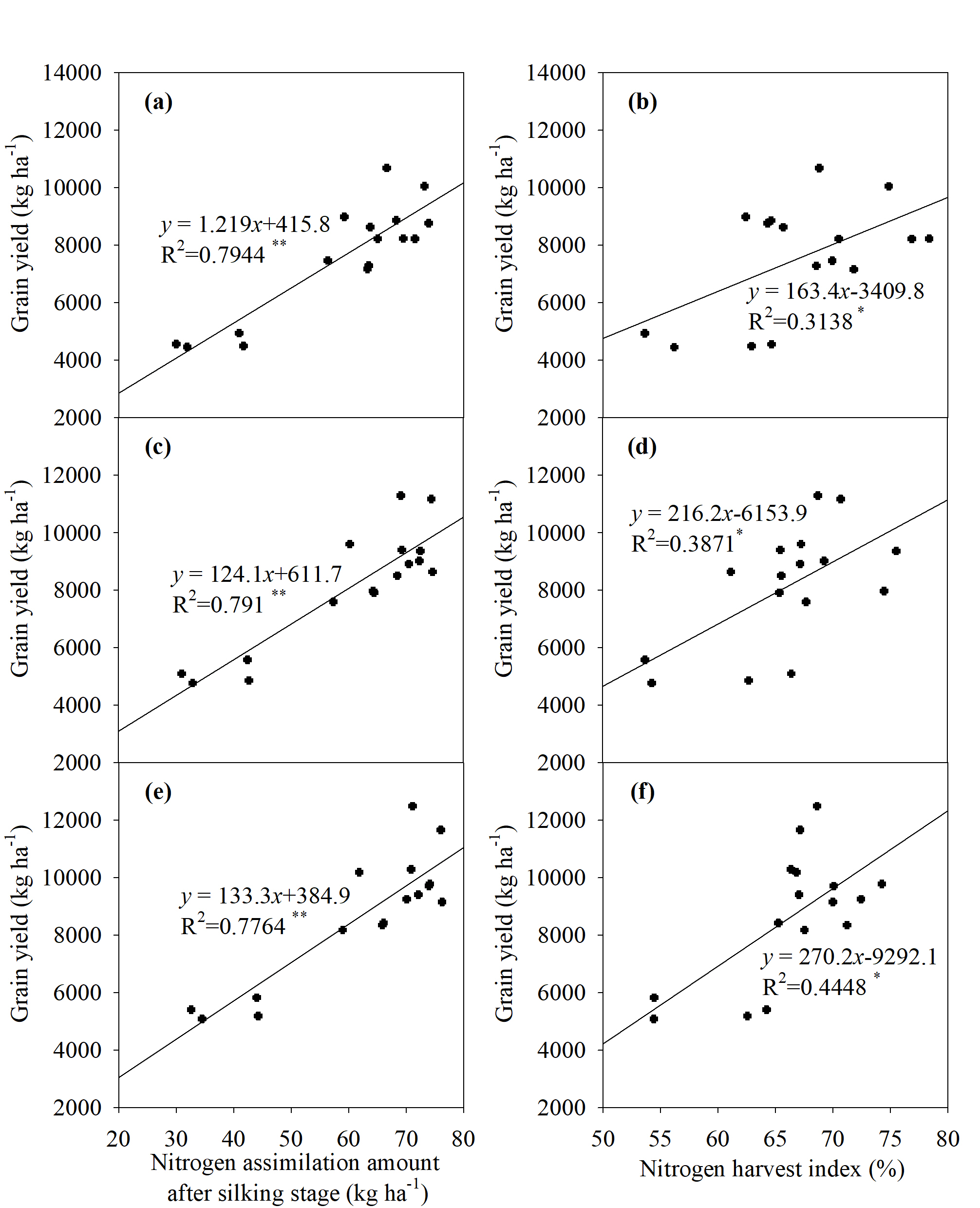


Figure S8. The relationship between nitrogen assimilation amount after silking stage and grain yield (a, c, e) and nitrogen harvest index and grain yield (b, d, f) in 2016 (a, b), 2017 (c, d), and 2018 (e, f). “**” means p < 0.01, “*” means 0.05 < p < 0.01 and “ns” means p > 0.05.


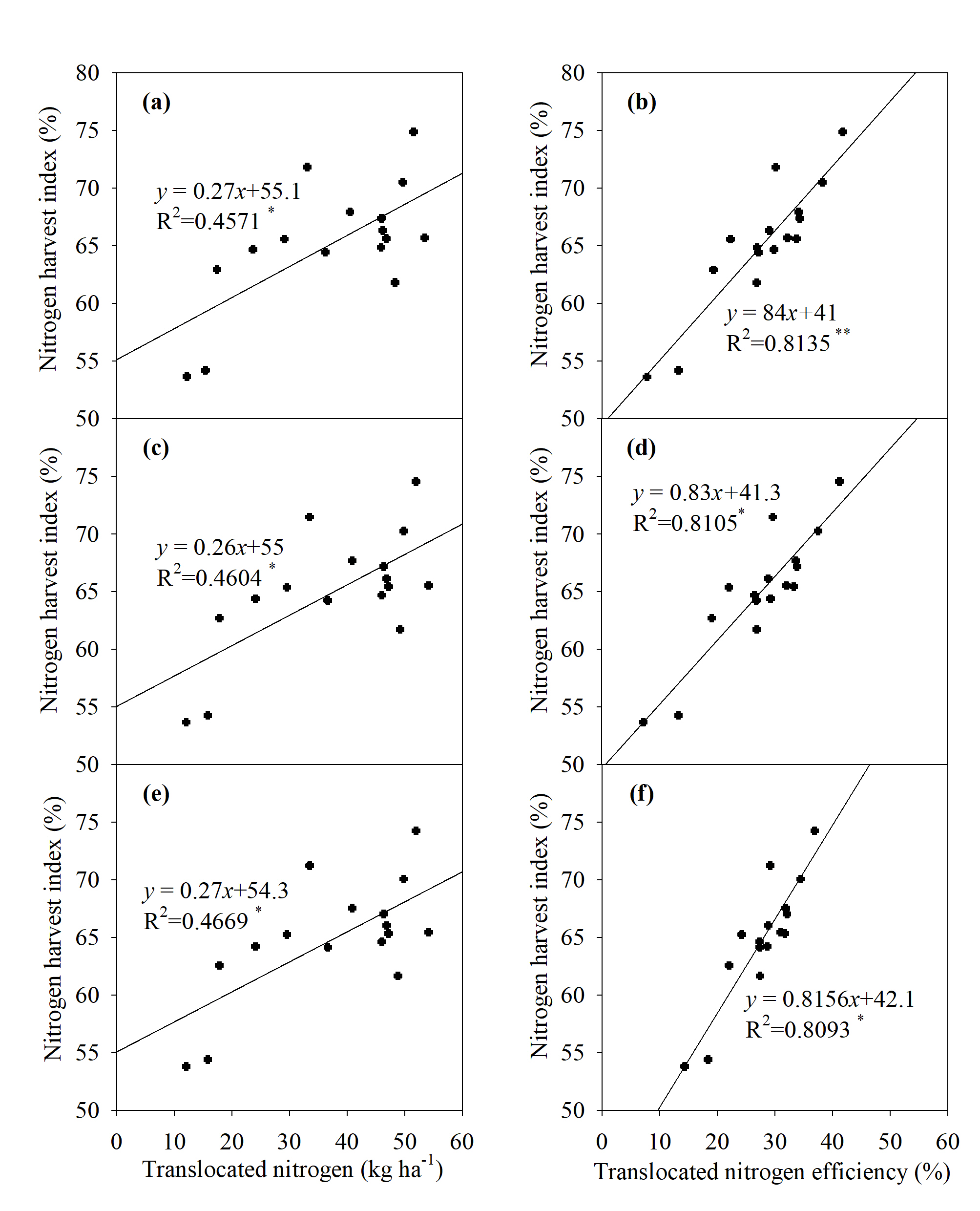


Figure S9. The relationship between translocated nitrogen and nitrogen harvest index (a, c, e) and translocated nitrogen efficiency and nitrogen harvest index (b, d, f) in 2016 (a, b), 2017 (c, d), and 2018 (e, f). “**” means p < 0.01, “*” means 0.05 < p < 0.01 and “ns” means p > 0.05.


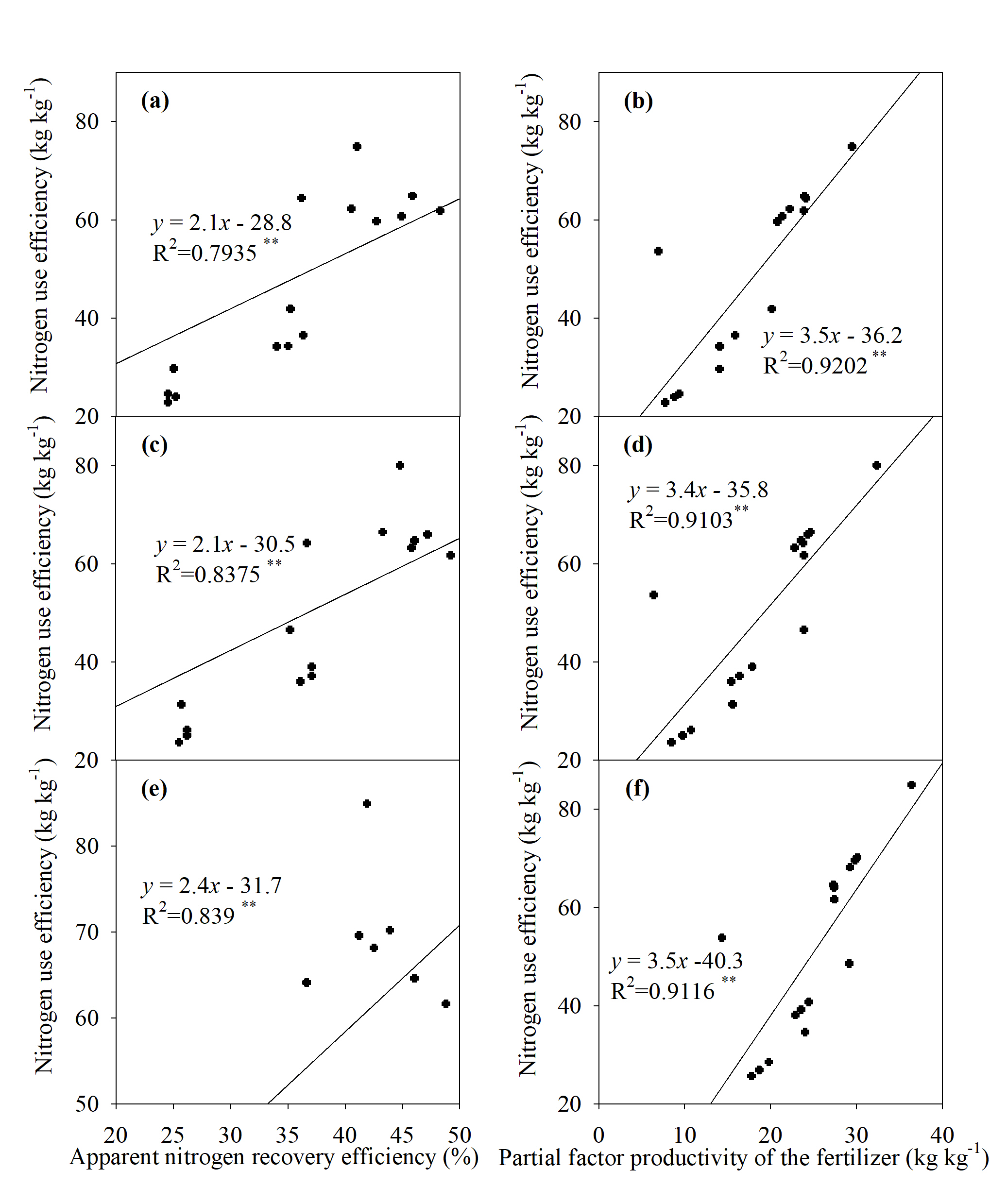


Figure S10. The relationship between nitrogen use efficiency and apparent nitrogen use efficiency (a, c, e) and nitrogen use efficiency and partial factor productivity of the fertilizer (b, d, f) in 2016 (a, b), 2017 (c, d), and 2018 (e, f). “**” means p < 0.01, “*” means 0.05 < p < 0.01 and “ns” means p > 0.05.
